# Optimizing the Classic and the Energy-Efficient Permutation Flowshop Scheduling Problem with a Hybrid Tyrannosaurus Rex Optimization Algorithm

**DOI:** 10.3390/biomimetics11040262

**Published:** 2026-04-10

**Authors:** Maria Tsiftsoglou, Yannis Marinakis, Magdalene Marinaki

**Affiliations:** School of Production Engineering and Management, Technical University of Crete, 73100 Chania, Greece; mtsiftsoglou@tuc.gr (M.T.); imarinakis@tuc.gr (Y.M.)

**Keywords:** Hybrid Tyrannosaurus Rex Optimization Algorithm (Hybrid TROA), permutation flowshop scheduling problem, comparative study, non-parametric statistical analysis, balance optimization, energy efficiency

## Abstract

This paper introduces a Hybrid Tyrannosaurus Rex Optimization Algorithm (Hybrid TROA) combined with Variable Neighborhood Search (VNS), two variations of the Path Relinking strategy, and a randomized Nawaz–Enscore–Ham (NEH) heuristic to address the Permutation Flowshop Scheduling Problem (PFSP). The TROA is a novel bio-inspired meta-heuristic algorithm modeled on the hunting behavior of the prehistoric Tyrannosaurus Rex. Leveraging the potential of this newly developed and efficient algorithm, we propose a framework in which an initial population of solutions is generated using the randomized NEH heuristic. These solutions are then further optimized through VNS and Path Relinking, yielding highly satisfactory results for the PFSP. First, we consider two optimization criteria separately: the makespan and the total flow time. Next, we conduct a comparative study of the Hybrid TROA against other prominent meta-heuristics, along with a statistical analysis using non-parametric tests, to determine the best-performing method for each objective. According to our findings, the Hybrid TROA proves to be the most suitable method in this study for minimizing both targets. Finally, recognizing that contemporary industry demands both high productivity and energy efficiency, we propose an energy-efficient version of the classic PFSP, simultaneously considering two criteria for optimization: the makespan and total energy consumption. Our study introduces a novel objective function that achieves balanced optimization by integrating both criteria.

## 1. Introduction

The Tyrannosaurus Rex Optimization Algorithm (TROA) is a novel meta-heuristic optimization method introduced by Sahu et al. [1]. The algorithm is inspired by the characteristics of the Tyrannosaurus Rex (T-Rex), a fearsome predator that lived in western North America over 66 million years ago during the Late Cretaceous period. The T-Rex is renowned for being one of the largest carnivores to ever walk the Earth, known for its immense bite force, sharp teeth, and advanced hunting skills. The TROA framework leverages these traits, simulating the T-Rex’s behaviors to navigate complex optimization landscapes effectively, demonstrating robust performance in finding optimal solutions. Sahu et al. [1] tested the algorithm on 24 benchmark problems, comparing its performance against seven well-known optimization techniques, including Differential Evolution (DE) [2], Particle Swarm Optimization (PSO) [3], and the Grey Wolf Optimizer (GWO) [4]. The results demonstrated the TROA’s superior performance in many cases. Later, the TROA drew the attention of researchers who aimed to enhance the original method. Zheng et al. [5] developed an improved TROA (ITROA) by utilizing the logistic chaotic mapping method and by incorporating the Golden Sine algorithm. The ITROA was tested against PSO, the GWO, the Whale Optimization Algorithm (WOA) [6], Dung Beetle Optimization [7], and the TROA on single-modal and multi-modal benchmark test functions, proving its superior performance. Zhang et al. [8] proposed a dynamic hierarchical improved TROA (DHTROA) with Hybrid Topology. The performance of the DHTROA was evaluated using the CEC2017 test functions and compared against nine other optimization algorithms, namely Pigeon-Inspired Optimization [9], the Cuckoo Optimization Algorithm [10], the Sine Cosine Algorithm [11], the WOA, Gorilla Troops Optimization [12], the Butterfly Optimization Algorithm [13], and the TROA. Additionally, the DHTROA was applied to six engineering optimization problems of varying complexity, demonstrating satisfying results.

A recent application of the TROA includes its integration with Support Vector Machines (SVMs). Specifically, Lei et al. [14] applied the TROA in conjunction with SVMs to enhance fault detection in wind turbine blades. By optimizing SVM parameters using the TROA, the method improved the efficiency and accuracy of fault detection, contributing to more reliable wind energy systems.

Despite the limited number of applications utilizing the TROA, its potential and efficiency in tackling optimization problems are evident. According to the authors’ knowledge, this is the first time the TROA has been employed in the field of scheduling. The PFSP [15,16] is widely encountered in industries like automotive, electronics, and textiles, where products must pass through multiple machines or stages in a specific sequence. The objective is to minimize the total processing time, commonly referred to as the makespan [17] or the total flow time [18]. Minimizing the makespan becomes increasingly important as the problem’s size grows. The PFSP is classified as an NP-hard problem [19], meaning that as the dimensions of the problem increase, the computational time required to find an optimal solution grows exponentially. Given these considerations, there is always a demand for effective methods to tackle the PFSP efficiently and promptly.

In our study, we propose a Hybridized TROA incorporating Variable Neighborhood Search (VNS) [20] and Path Relinking strategies [21] to tackle the PFSP. Two objective values are considered for minimization: the makespan and the total flow time. To achieve even more satisfactory results, a randomized Nawaz, Enscore, and Ham (NEH) [22] heuristic is employed for generating the initial population of solutions.

To evaluate effectiveness of the Hybrid TROA, we compare it against partial hybrid variations, namely TROA-VNS-PR, TROA-NEH-PR, as well as the classical TROA. The results demonstrate that the integration of all hybridization components leads to a greater performance improvement over the classical TROA than the other tested variants.

The ITROA and DHTROA are two high-performing hybrid variants of the classical TROA. For the purposes of this study, both variants were appropriately adapted to solve the PFSP and were subsequently compared with the proposed Hybrid TROA. The results suggest that the proposed method is better suited to tackling the PFSP than the ITROA and DHTROA, since it was specifically designed for this problem, whereas the other two variants were originally developed for different purposes.

In addition, the Hybrid TROA is tested against eight other prominent meta-heuristics, namely PSO, the GWO, the WOA, Artificial Bee Colony (ABC) [23], the Bat Algorithm (BA) [24], Tuna Swarm Optimization (TSO) [25], the Sparrow Search Algorithm (SSA) [26], and the Firefly Algorithm (FA) [27], utilizing Taillard’s [28] benchmark datasets for the makespan criterion. All meta-heuristics were developed within the same framework as the Hybrid TROA. To evaluate their performance, we conducted a statistical analysis, including box plots and non-parametric tests such as Friedman’s test, Friedman’s Aligned test, the Quade test, and p-adjusted comparison tests.

For the Energy-Efficient PFSP, its classic version is modified to allow for different speed rates for each machine. The speed rate influences both the minimization of the makespan and the simultaneous increase in energy consumption. Two categories of decision variables are considered: the first pertains to the permutation of jobs and the second to the speed rate of each machine. The authors created an objective function in order to obtain balance between the makespan and energy consumption. As solution methods, two nature-inspired meta-heuristics are proposed: the Hybrid TROA and the Hybrid GWO. Additionally, computational experiments on benchmark datasets are conducted to evaluate the effectiveness of each method in addressing the Energy-Efficient PFSP by optimizing the balance between the makespan and energy consumption.

This paper is organized as follows: Section 2 presents the definition of the Permutation Flowshop Scheduling Problem, while Section 3 examines the main characteristics of the TROA detailing each method presented, its purpose, and how it functions. In Section 4, a comparative study is conducted between the Hybrid TROA and other TROA variants and several meta-heuristics algorithms, together with a comprehensive statistical analysis aimed to determine whether any algorithm exhibits statistically significant superior performance. Finally, Section 5 presents the main characteristics of the Energy-Efficient PFSP and a proposed objective function, along with a comparative analysis between the Hybrid TROA and Hybrid GWO, while Section 6 provides the conclusions and the future research perspectives.

## 2. Problem Definition

### The Permutation Flowshop Scheduling Problem

The Permutation Flowshop Scheduling Problem (PFSP) can be mathematically formulated as an optimization problem. Let us denote the set of *n* jobs as *N* = {1, 2, …, *n*} and the set of *m* machines as *Μ* = {1, 2, …, *m*}. The objective is to find a permutation *π* = {*π*_1_, *π*_2_,…, *π*_*n*_} of the jobs for each machine that minimizes the makespan (C_max_). The basic assumptions for the PFSP are as follows:

- The sequence of jobs must be processed on the machines in the same order, i.e., from machine 1 to machine *m*.
- Each machine must process the jobs according to the given permutation.
- No pre-emption is allowed, meaning the processing of a job $\pi_{i}$ on the $j^{th}$ machine cannot be interrupted.
- All jobs are independent and available for processing at time zero.
- Each job is restricted to being processed on a single machine at any given time, and similarly, each machine can process only one job.
- Set-up times for jobs on machines are negligible and, therefore, can be disregarded.
- Furthermore, the machines are continuously available and waiting for the next operation.

The transportation time for delivering one job between the machines is neglected.

According to a certain π permutation of jobs, the job $\pi_{i}$ is placed at the $i^{th}$ position, while $C_{\pi_{i}j}$ denotes the completion time of that job on the machine *j*. The variable $p_{\pi_{i}j}$ denotes the processing time for the job $\pi_{i}$ on the *j* machine. The mathematical description of the problem is provided in the following lines:

(1) $$C_{\pi_{1},1}=\;p_{\pi_{1,}1}$$

(2) $$C_{\pi_{i},1}=C_{\pi_{i-1},1}+p_{\pi_{i},1}\;\forall i=2,\ldots ,n$$

(3) $$C_{\pi_{1,}j}=C_{\pi_{1},j-1}+p_{\pi_{1},j}\;\forall j=2,\ldots ,m$$

(4) $$C_{\pi_{i},j}=\max\left\{C_{\pi_{i-1},j},C_{\pi_{i},j-1}\right\}+p_{\pi_{i},j}\;\forall i=2,\ldots ,n;\forall j=2,\ldots ,m$$

The makespan of the permutation π is defined as the completion time of the last job $\pi_{n}$ on the last machine *m*, i.e.,

(5) $$C_{max}\left(\pi \right)=C(\pi_{n},m)$$

Therefore, the PFSP with the makespan criterion is to find the optimal permutation$\;\pi^{*}$ in the set of all possible permutations Π such as:

(6) $$C_{max}\left(\pi^{*}\right)\leq C\left(\pi_{n},m\right)\;\forall \pi \in \Pi$$

As for the total flow time criterion, let $F(\pi_{i}$) represent the flow time of the job $\pi_{i}$. Clearly, $F(\pi_{i}$) is equivalent to the completion time $C_{\pi_{i},m}\;$ of the job $\pi_{i}$ on the last machine *m*, since the release times of all jobs are zero. The total flow time $TFT(\pi_{i})$ of a permutation $\pi_{i}\;$can be computed by summing the flow times or completion times of all jobs. Then, the total flow time of a permutation $\pi_{i}$ is defined as:

(7) $$TFT(\pi_{i})=\;\sum_{i=1}^{n}F(\pi_{i})\;=\;\sum_{i=1}^{n}C_{\pi_{i},m}$$

Therefore, the Permutation Flowshop Scheduling Problem with the total flow time criterion is to find the optimal permutation $\pi^{*}$ in the set of all permutations $\pi_{i}$ such that:

(8) $$TFT\left(\pi^{*}\right)\leq TFT\;\left(\pi_{i}\right)\;\forall \pi \in \Pi$$

## 3. The Hybrid TROA with VNS and Path Relinking Strategy

### 3.1. Inspiration and Key Formulations of TROA

The TROA is a population-based algorithm in which the individuals represent the prey and the target among the crowd represents the optimal solution. Let $X_{i}^{t}=[x_{i1}^{t},x_{i2}^{t},\ldots ,x_{id}^{t}]$ denote the location of the i-th prey at iteration t, where i=1, 2,…,N refers to the individuals of the population, N is the population size, and d is the dimension of the problem.

The T-Rex hunts randomly and targets the nearest prey for a strike. The prey, however, may either defend itself or escape if it is quick enough. This hunting behavior, along with the prey’s probability of escaping, is mathematically represented by the following equation:

(9) $$\begin{array}{c} X_{i}^{new}=\left\{\begin{array}{cc} X_{new}, & \mathrm{if}\;rand()<E_{r}\\ X_{rand}, & \mathrm{otherwise} \end{array}\right. \end{array}$$

where $E_{r}$ is the estimation of reaching the scattered prey, rand() generates a uniformly distributed random number in the interval [0, 1], and $X_{rand}$ denotes a randomly generated position vector within the search space.

As soon as the prey detects the presence of the T-Rex, it starts running and scatters. The T-Rex then adjusts its position according to the following equation:

(10) $$X_{new}=X_{i}^{t}+rand()\cdot sr\cdot \left(T^{\;t}\cdot tr-{Target}_{i}^{\;t}\cdot pr\right)$$

where sr is the success rate, defined in the interval [0.1, 0.9], tr is the T-Rex’s running rate, defined in the interval [0.067, 0.3], and pr is the prey’s running rate, defined in the interval [0.1, 0.9]. Moreover, $T^{\;t}$ denotes the current position of the T-Rex at iteration t, while ${Target}_{i}^{\;t}$ denotes the target position associated with the i-th prey. If sr=0, the prey is assumed to have escaped, the hunting attempt fails, and the corresponding target position is set to zero.

The next step is the selection process, which depends on the comparison between the prey’s previous position and its updated position. Thus, the new position of the i-th prey is determined as follows:

(11) $$X_{i}^{t+1}=\left\{\begin{array}{cc} X_{i}^{new}, & \mathrm{if}\;f(X_{i}^{new})<f(X_{i}^{t})\\ X_{i}^{t}, & \mathrm{otherwise} \end{array}\right.$$

where $f(X_{i}^{t})$ is the fitness value of the prey’s current position, and $f(X_{i}^{new})$ is the fitness value of its updated position. For minimization problems, the position with the lower fitness value is retained. In Algorithm 1, a pseudocode of the TROA is given:
**Algorithm 1. T-REX Optimization Algorithm (TROA) pseudocode**      1. **Initialize** the prey positions $X_{i}^{0}$, i=1, 2,…,N, randomly.      2. Initialize the max number of iterations $Iter_{max}$.      3. Initialize parameters Er, pr,tr, sr.      4. **Set** the iteration counter t=0.      5. **Evaluate** the fitness values $f(X_{i}^{t})$ for all prey positions.      6. **Determine** the best prey position and set it as the target.      7. **While** $t<Iter_{max}$
**do**       8.    **Move** the T-Rex randomly.       9.    **For** each prey i=1, 2,…,N
**do**
      10.    **If** $rand()<E_{r}$
**then**
      11.     **Generate** $X_{i}^{new}$ using Equation (10).       12.   **Else**        13.     **Generate** $X_{i}^{new}$ randomly.       14.   **End If**      15.   **Evaluate** $f(X_{i}^{new})$.      16.   **Update** $X_{i}^{t+1}$ and the target according to Equation (11).      17.  **End For**      18.  **Determine** the best solution found so far.      19.  **Set**
t=t+1.      20. **End While**      21. **Return** the best solution found.

### 3.2. Generating the Initial Population of Solutions

Bio-inspired algorithms utilize a population of solutions and, through an iterative process, exchange and develop new solutions. These new solutions are evaluated based on a specific optimization criterion. If a new solution has a better fitness value, the previous solution is discarded, and the improved solution takes its place. Often, the initial population is generated randomly within specific boundaries, ensuring diversity in the solutions and preventing premature convergence. On the other hand, a completely random initial population may result in poor coverage of the search space, thus reducing the chances of finding high-quality solutions. A combination of a constructive heuristic algorithm and a random element address the above issue by providing a diverse sample of feasible, good quality solutions.

In this paper, a randomized NEH heuristic with the triangular distribution is utilized for generating the initial population of solutions. The triangular distribution has three parameters: the minimum (a), the maximum (b), and the mode (c). We set these parameters according to the following formulations:

(12) $$a=\min\left\{\sum_{j=1}^{m}p_{\pi_{1}j},\sum_{j=1}^{m}p_{\pi_{2}j},\ldots ,\sum_{j=1}^{m}p_{\pi_{n}j}\right\}$$

(13) $$b=\max\left\{\sum_{j=1}^{m}p_{\pi_{1}j},\sum_{j=1}^{m}p_{\pi_{2}j},\ldots ,\sum_{j=1}^{m}p_{\pi_{n}j}\right\}$$

(14) $$c=\frac{\alpha }{b}$$

The triangular distribution determines the probability of a job being selected next in the permutation, considering its completion time. Seventy percent (70%) of the initial solutions are generated using the randomized NEH, ensuring diversity along with good quality. The remaining 30% are generated randomly, thereby including solutions from a broader search space.

### 3.3. Encoding and Decoding

The formulations of the TROA represent solutions in a continuous search space. Since the PFSP requires a solution vector in discrete form (the permutation of jobs), an encoding–decoding technique is adopted. In the case of a continuous solution vector, we map the smallest value to job number 1, the next smallest value to job number 2, and continue this process until all values in the vector are transformed into the discrete form. For instance, given $\vec{X_{c}}=[\;0.1434\;,\;-0.3456\;,\;2.567]$, the transformation proceeds step by step: $\vec{X_{c}}=[\;0.1434,\;1,\;2.567]$, then $\vec{X_{c}}=[\;2,\;1,\;2.567]$, and finally $\vec{X_{d}}=[2,\;1,\;3]$. If we want to transform the discrete solution vector $\vec{X_{d}}$, we divide each individual value in the vector by the greatest value in the vector.

### 3.4. The Proposed Variable Neighborhood Search Algorithm

The Variable Neighborhood Search (VNS) is a meta-heuristic procedure that explores the solution space in the vicinity of a current solution by applying one or more neighborhood operators. In the present study, each current solution is denoted by $X_{i}^{t}$, while the corresponding candidate solution generated by the VNS procedure is denoted by $X_{i}^{new}$. At each iteration, VNS applies a randomly selected neighborhood operator to $X_{i}^{t}$ in order to generate $X_{i}^{new}$ and evaluates its quality through the objective function $f(X_{i}^{new})$. If the candidate solution improves the current one, that is, if $f(X_{i}^{new})<f(X_{i}^{t})$, then the current solution is updated accordingly. In this study, the neighborhood operators employed are 2-opt, 3-opt, 1–0 relocate, 2–0 relocate, 1–1 exchange, and 2–2 exchange. The proposed VNS procedure is applied to each solution in the population, with the neighborhood operator being selected randomly at each iteration. If no improvement is observed for a specified number of consecutive attempts, tracked by the no-improvement counter c, the neighborhood operator is randomly changed, and the search process continues. In the present implementation, the neighborhood change is triggered when c=10. The proposed VNS procedure is described in Algorithm 2.
**Algorithm 2. Variable Neighborhood Search (VNS) pseudocode**      1. **Initialize** the iteration counter t=0.      2. Initialize the no-improvement counter c=0.      3. **Determine** the best solution in the population and set it as $X_{best}$.      4. **While** the stopping criterion is not met **do**
      5.  **Select** a solution $X_{i}^{t}$ randomly from the population.      6.  **Generate** a random integer $Counter_{VNS}\in \{1,\;2,\;3,\;4,\;5,\;6\}$.      7.  **Generate** a candidate solution $X_{i}^{new}$ by applying the corresponding neighborhood operator:      8.     **If** $Counter_{VNS}=1$, **apply** the 2-opt operator.      9.     **Else if** $Counter_{VNS}=2$, **apply** the 3-opt operator.      10.   **Else if** $Counter_{VNS}=3$, **apply** the 1–0 relocate operator.      11.   **Else if** $Counter_{VNS}=4$, **apply** the 2–0 relocate operator.      12.   **Else if** $Counter_{VNS}=5$, **apply** the 1–1 exchange operator.      13.   **Else if** $Counter_{VNS}=6$, **apply** the 2–2 exchange operator.      14.  **End If**      15.  **Evaluate** the fitness value $f(X_{i}^{new})$.      16.  **If** $f(X_{i}^{new})<f(X_{i}^{t})$ **then**
      17.   **Set** $X_{i}^{t}=X_{i}^{new}$.      18.   **If** $f(X_{i}^{t})<f(X_{best})$ **then**
      19.     **Set** $X_{best}=X_{i}^{t}$.       20.   **End If**
      21.   **Set** c=0.       22.  **Else**
      23.   **Set**
c=c+1.      24.  **End If**       25.  **Set** t=t+1.      26. **End While**
       27. **Return** $X_{best}$.

### 3.5. Two Variations of the Path Relinking Strategy

In the context of this study, two variations of the Path Relinking strategy are developed. Path Relinking is a procedure in which two solutions are used to construct a new solution that shares characteristics with both. One solution serves as the starting point, denoted by $X_{start}$, while the other acts as the target solution, denoted by $X_{target}$. Starting from $X_{start}$, the method incrementally applies relocate and exchange moves to transform the current solution into $X_{target}$. At each step, an intermediate solution is generated, lying between the starting and target solutions. These intermediate solutions may provide better objective values than the original ones.

In the first version of Path Relinking, any solution in the population may serve as $X_{start}$, whereas the best solution in the population is used as $X_{target}$. This version is applied sequentially to all solutions in the population, except the global best solution. In the second version, two solutions are randomly selected from the population: one is assigned as $X_{start}$ and the other as $X_{target}$. This process is repeated for a predefined number of iterations. A pseudocode of the first version of Path Relinking is presented in Algorithm 3.
**Algorithm 3. Path Relinking (First Version) pseudocode**      1. **Set** $X_{start}$ as the start solution.      2. **Set** $X_{target}\;$as the best-known solution.      3. **Set** $X_{current}=X_{start}$.      4. **Set** $X_{best}=X_{current}$.      5. **While** $X_{current}\neq X_{target}$ **do**      6. **Identify** the set of moves M required to transform $X_{current}$ into $X_{target}$.      7. **Evaluate** all possible moves in M in order to generate neighboring solutions.      8. **Select** the best move $m_{best}\in M\;$according to the objective function f(⋅).      9. **Apply** $m_{best}\;$to $X_{current}$ to generate the next solution.      10. **Set** $X_{current}$ equal to the generated next solution.      11. **If** $f(X_{current})<f(X_{best})$ **then**      12. **Set** $X_{best}=X_{current}$.      13. **End If**      14. **End While**      15. Return $X_{best}$.

The pseudocode is the same for the second version, except for the selection of the starting and target solutions, which happens randomly.

### 3.6. Flowchart of the Proposed Solution Method

**Figure 1:**
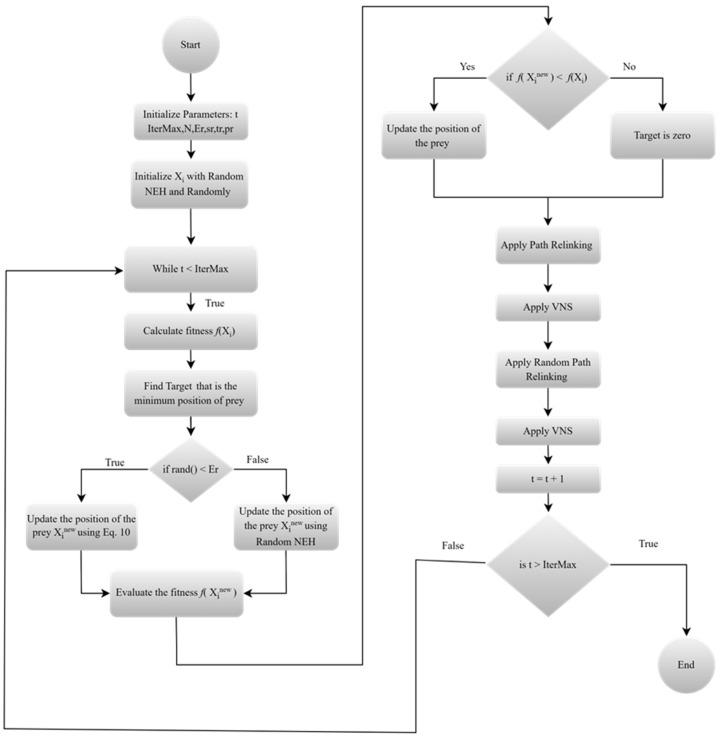
Flowchart of the proposed Hybrid TROA method.

The flowchart of the proposed Hybrid TROA is presented in Figure 1.

The stopping criterion was defined by setting the maximum number of iterations IterMax equally. The initial population consisted of N individuals and was generated according to Section 3.2. At each iteration, the fitness of all solutions was evaluated with respect to the selected objective function, namely the makespan or the total flow time criterion, and the best solution was selected as the Target. Then, for each solution $X_{i}^{t}$, if rand(), a uniformly distributed random number in [0, 1], was smaller than $E_{r}$, the solution was updated according to Equation (10); otherwise, $X_{i}^{new}$ was generated using the randomized NEH procedure. The fitness value $f\left(X_{i}^{new}\right)\;$ was then computed, and whenever $f(X_{i}^{new})<f(X_{i}^{t})$, the solution was updated as $X_{i}^{t+1}=X_{i}^{new}$. Otherwise, the Target was set to zero. Afterwards, all solutions were further refined through the proposed local search scheme. The overall procedure was repeated until the iteration counter reached IterMax.

### 3.7. Computational Complexity

The computational complexity of the proposed Hybrid TROA depends on several factors, including the number of jobs n, the number of machines m, the population size P, and the number of iterations T. The evaluation of a single PFSP solution (i.e., a permutation of jobs) requires computing the makespan, which has a time complexity of

O(n⋅m)

For each iteration, the algorithm processes all individuals in the population. Thus, the cost of solution evaluation per iteration is:

O(P⋅n⋅m)

Additionally, the exploration phase involves generating new candidate solutions randomly or based on Equation (10), which have a complexity of approximately:

O(P⋅n)

A significant portion of the computational effort arises from the embedded Variable Neighborhood Search procedure. Assuming that the VNS examines k neighboring solutions per individual, the corresponding complexity becomes:

O(P⋅k⋅n⋅m)

Therefore, the overall time complexity of the algorithm can be approximated as:

$$O\left(T\cdot P\cdot (n\cdot m+k\cdot n\cdot m)\right)$$

or equivalently:

O(T⋅P⋅k⋅n⋅m)

in the case where the local search dominates the computational cost. This analysis indicates that the algorithm scales linearly with respect to the number of iterations and population size and polynomially with respect to the problem dimensions n and m. However, the inclusion of VNS, while improving solution quality, increases the computational burden, especially for large-scale PFSP instances.

## 4. Computational Experiments and Hybrid TROA Evaluation Against Other Swarm Intelligence Meta-Heuristics

### 4.1. Parameter Analysis

**Figure 2:**
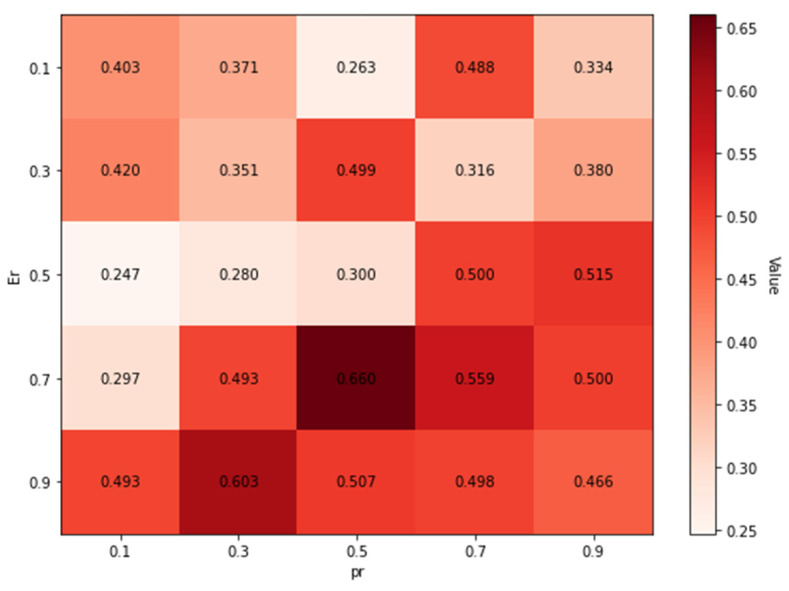
Parameter analysis of the Er and pr parameters for the Hybrid TROA based on the mean normalized best value (lower values indicate better performance).

The classical TROA achieves a balance between exploration and exploitation by means of four control parameters, namely $E_{r}$ (estimation of reaching the scattered prey), sr (success rate), pr (prey running rate), and tr (T-Rex running rate). Within the scope of this study, a parameter analysis was performed on six representative instances selected from the 60 Taillard benchmark instances considered in the experimental evaluation. In particular, the first instance of each of the six benchmark sets was selected (ta001, ta011, ta021, ta031, ta041, and ta051), as these instances correspond to different levels of computational difficulty. For each possible combination of $E_{r}\in [0.1,\;0.9]$ and pr∈[0.1, 0.9], the best makespan value was recorded after three independent runs on every instance. To account for the varying difficulty levels of the selected instances, the obtained mean values were normalized prior to aggregation. This normalization enabled a fair comparative assessment of the tested parameter combinations and the derivation of an overall relative ranking, which is presented in Figure 2.

According to the heat map presented in Figure 2, the parameter combination Er = 0.67 and pr = 0.25 was selected. The remaining parameters were set to tr=0.3 and sr = 0.8, as recommended in the literature [1].

As for the parameters of the remaining meta-heuristics, the recommended values reported in the literature were adopted. Specifically, in the Hybrid GWO and Hybrid WOA, the control parameter α was linearly decreased from 2 to 0 throughout the iterations. For Hybrid PSO, the acceleration coefficients $c_{1}$ and $c_{2}$ were both fixed at 2. In the Hybrid FA, the parameters β and γ were both set to 0.9. The parameters of the Hybrid SSA were set to $R_{2}=0.2$ and ST=0.6, while the producer-to-scrounger ratio was fixed at 75/25. The Hybrid BA involves two significant parameters, namely α and γ, which were both set to 0.96 in this study. Similarly, for the Hybrid ABC algorithm, two parameters were considered: the bee population size and the limit for employed bees. In our study, the bee population was set to 100, while the employed-bee limit was set to 1. Finally, for Hybrid TSO, the parameters z and a were set to 0.05 and 0.7, respectively.

### 4.2. Results for the Makespan Criterion

In order to evaluate the performance of the Hybrid TROA in solving the PFSP for the makespan ($C_{max}$) and total flow time (TFT) criteria, we utilize Taillard’s benchmark dataset. The datasets are different in terms of size and complexity. The first, second, and third sets include ten instances, each of 20 jobs and 5, 10, and 20 machines, respectively. The fourth, fifth, and sixth sets include ten instances, each of 50 jobs and 5, 10, and 20 machines, respectively.

The stopping criterion was defined by setting the maximum number of iterations IterMax equal to 20. The initial population consisted of 100 individuals and was generated 70% with randomized NEH and the remaining 30% randomly. For a total of sixty instances, we conducted ten runs employing the Hybrid TROA method for both criteria, the makespan and total flow time. The algorithms were implemented in Python 3.13, and all runs were executed on a Lenovo ThinkPad P1 workstation with Fedora Linux 43, an Intel Xeon E-2176M CPU at 2.70 GHz, and 32 GiB of RAM. No GPU acceleration was used.

In Table 1, N denotes the instance number, n represents the number of jobs, and m indicates the number of machines. The column BKS presents the best-known solution for the makespan, obtained so far for each specific instance. The column Hybrid TROA shows the best obtained value for the makespan achieved using the Hybrid TROA. AVG is the average value of ten runs with the Hybrid TROA, while St. Dev denotes the standard deviation calculated according to Equation (15). The column ME (%) presents the mean error percentage calculated according to Equation (16).

(15) $$St.Dev.=\sqrt{\frac{\sum {\left(x_{i}-\mu \right)}^{2}}{n}}$$

where $x_{i}$ represents each value of the population, μ denotes the population’s mean value, and n is the population size.

(16) $$Mean\;Error(ME)=\frac{C_{TROA}-C_{BKS}}{C_{BKS}}\%$$

**Figure 3:**
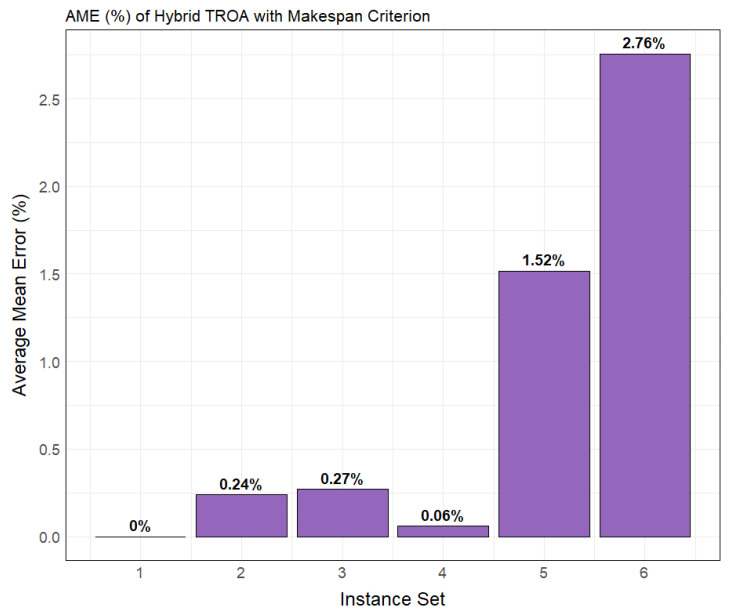
Bar plot illustrating the average mean error (%) of the Hybrid TROA for Taillard’s instance sets for the makespan criterion.

For a clearer comprehension of the Hybrid TROA’s performance in tackling the PFSP with makespan measure, Figure 3 portrays the average mean error (%) on the *y*-axis, calculated as per Equation (17), while the *x*-axis depicts the instance set. The Hybrid TROA demonstrates highly satisfactory average performance for sets 1 and 4, encompassing 20 × 5 (jobs and machines) and 50 × 5 (jobs and machines), respectively. For the datasets with the highest complexity, represented by the last two sets, Figure 3 indicates an increase in the average mean error.

(17) $$Average\;Mean\;Error(AME)=\frac{\sum_{i=1}^{N}ME_{i}}{N}$$

The average mean error (AME) is calculated by averaging the mean errors within each instance set. Since each set comprises 10 instances, the total mean error for the set is divided by 10 to obtain the AME. Additional analysis of the Hybrid TROA’s robustness and effectiveness is carried out in subsequent sections, accompanied by statistical examination.

**Figure 4:**
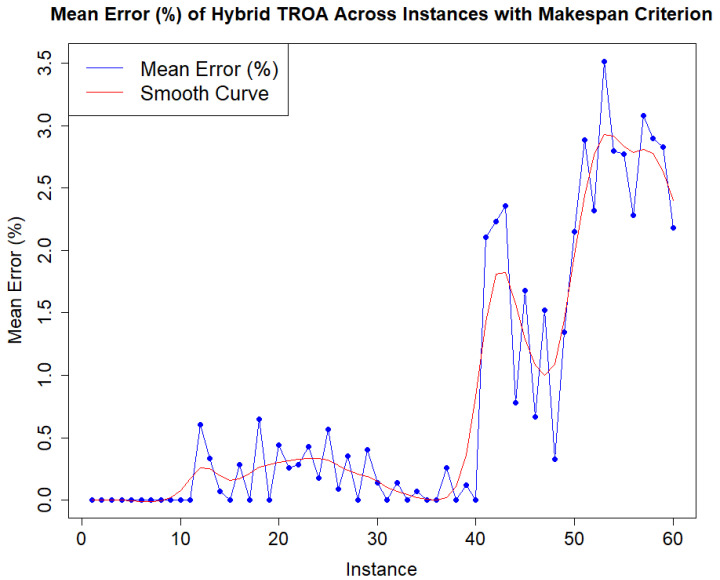
Hybrid TROA’s mean error progress of makespan criterion for each instance.

**Figure 5:**
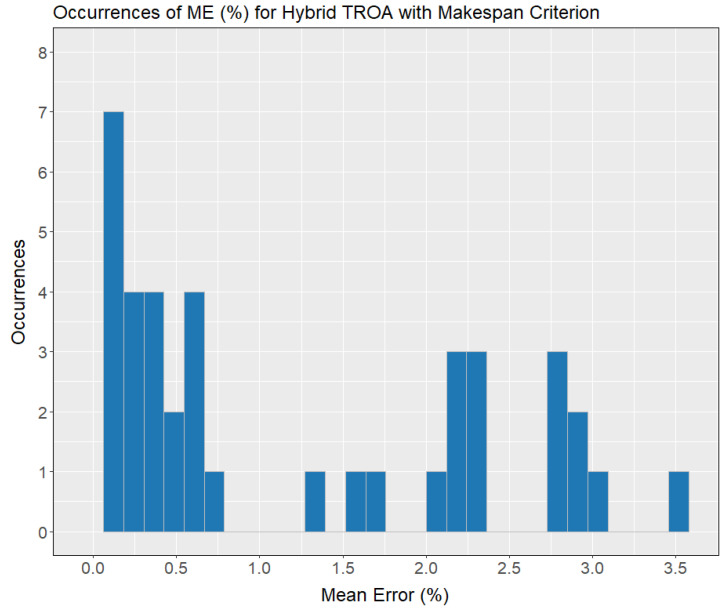
Occurrences of mean error (%) for the makespan criterion using the Hybrid TROA method.

Figure 4 explicitly illustrates the progress of mean error with increasing instance complexity. The data show that the mean error remains relatively stable, with a slight increase between the second and third instance groups, followed by a decline up to the fourth instance group. Beyond this point, the mean error escalates notably in the last two, more complex sets, as expected. In Figure 5, the occurrences of mean errors (%) for the Hybrid TROA is clearly depicted. The majority of errors, 43 out of 60 (71.67%), fall between 0 and 1%, while 35% have zero mean error value. Three out of sixty mean errors (5%) are between 1% and 2%. Twelve out of sixty (20%) fall within the range of 2% and 3%, while the remaining 3.33% of mean errors lie between 3% and 4%. The maximum mean error (%) value observed is 3.52 in the instance number 53.

### 4.3. Results for the Total Flow Time Criterion

The TFT criterion represents the sum of the completion times of all jobs in a given schedule. Lower TFT values indicate more efficient schedules in terms of minimizing job completion delay. It is a crucial metric when focusing on reducing the gaps between jobs. Table 2 presents the results of the TFT criterion for the schedules created using the Hybrid TROA method. In order to evaluate our method, we once again utilize the Taillard benchmark datasets.

**Figure 6:**
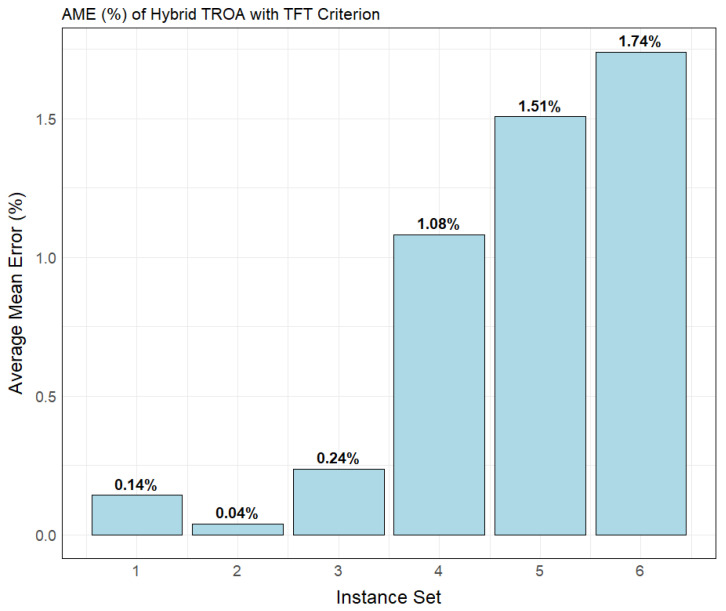
Bar plot illustrating the average mean error (%) of the Hybrid TROA for Taillard’s instance sets for the TFT criterion.

Figure 6 presents the AME (%) for each instance set with the TFT criterion. The second instance set has the lowest AME (%), equal to 0.04%, followed by the first instance set at 0.14%. The third instance set shows a small increase in AME (%), with a value of 0.24%. The last three sets, due to their higher complexity, show a progressive increase in the AME (%) measure, which is expected.

**Figure 7:**
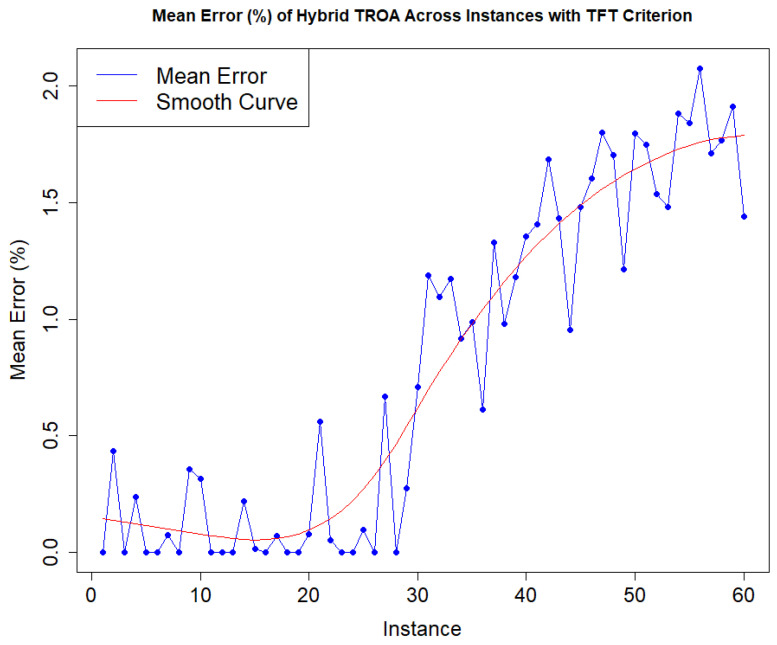
Hybrid TROA’s mean error progress of total flow time criterion for each instance.

Figure 7 illustrates the mean error (ME%) values for each instance using the TFT criterion. The first 30 instances remain relatively low with some fluctuations. However, in the later instances of the second set, the data displays an exponential growth pattern. The problem dimensions in the later datasets progressively increase, which justifies the corresponding rise in ME (%) values.

**Figure 8:**
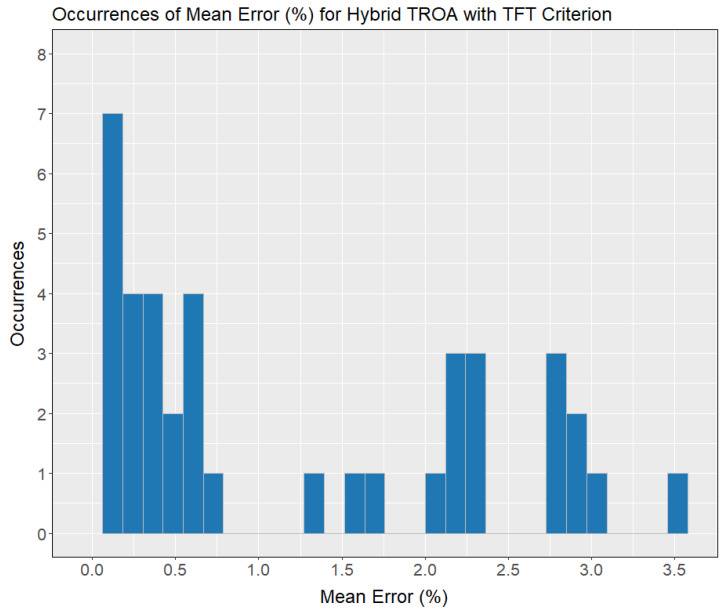
Occurrences of mean error (%) for the total flow time criterion using the Hybrid TROA method.

Figure 8 shows the distribution of ME (%) based on the TFT criterion. Twelve out of sixty instances (20%) fall within the range of 0% to 0.5% ME. Six out of sixty instances (10%) have ME values between 0.5% and 1%. Another 20% of instances fall within the 1% to 1.5% ME range. Additionally, 14 out of 60 instances (23.33%), which represents the highest concentration, fall within the 1.5% to 2% ME range. The highest ME value, 2.08%, is observed in a single instance out of 60.

### 4.4. Comparative Analysis of Hybrid TROA Against Other TROA Variations

In order to compare the performance of the proposed Hybrid TROA, we conducted a comparative analysis with other TROA variations, thus establishing whether the Hybrid TROA is a competitive method. First, the classical TROA with a random initial population was extended to solve the PFSP. The second variation is TROA-NEH-PR, in which the classical TROA is hybridized with the proposed randomized NEH and the proposed Path Relinking. The third variant is TROA-VNS-PR, in which the original algorithm is hybridized with the proposed VNS and Path Relinking. The algorithms were tested on Taillard’s benchmark datasets; specifically, the first instance of each of the first six set was selected for the experiments. For every method and every instance, ten independent runs were executed, and the best overall mean error was recorded. Table 3 presents comparative performance of the Hybrid TROA against the TROA, TROA-NEH-PR, and TROA-VNS-PR in terms of average mean error (AME). The Hybrid TROA achieves the best overall performance, with the lowest AME of 0.88, outperforming all other variations in across all testing instances. This shows that full hybridization, that is, combining randomized NEH initialization, Path Relinking, and VNS, is the most effective configuration. It suggests that these three components are complementary. More specifically, TROA-NEH-PR performs better than the classical TROA, indicating that NEH-based initialization and Path Relinking improve the original algorithm. In contrast, TROA-VNS-PR is outperformed by the classical algorithm, suggesting that VNS and Path Relinking alone are not sufficient to improve the search. This is an important finding, because it highlights that for the TROA, the quality of initial solutions appears to play a more critical role the local search improvement. Overall, the results indicate that the initialization mechanism plays a particularly important role, while the full cooperation of all hybrid components leads to more robust and competitive variant.

Since the Hybrid TROA appears to be the most competitive hybrid variation, a comparative analysis is further conducted against two improved TROA variants from the literature, namely the ITROA and DHTROA. As the ITROA employs a chaotic initialization mechanism, whereas the DHTROA adopts a chaotic opposition-based initialization strategy, we chose not to modify their original initialization schemes. Instead, in order to preserve the original design principles of these methods while ensuring a fair comparison, we only incorporated the proposed VNS and Path Relinking used in the Hybrid TROA. The algorithms were tested on Taillard’s benchmark datasets; specifically, the first instance of each of the first six sets was selected for the experiments. For every method and every instance, ten independent runs were executed, and the overall mean error was recorded. Table 4 reports the comparative performance of the Hybrid TROA against the ITROA and DHTROA in terms of average mean error (AME). The results indicate that the Hybrid TROA provides the most favorable overall performance, achieving the lowest AME value (0.88) compared with the ITROA (1.78) and DHTROA (2.74).

The observed results may be attributed to the different roles of the hybrid components in the tested algorithms. Although the ITROA and DHTROA were enriched with the same VNS and Path Relinking framework in order to ensure a fair comparison with the Hybrid TROA, the latter retains an important advantage through its NEH-based initialization mechanism. Since NEH is specifically tailored to permutation scheduling problems, it provides high-quality initial solutions that are particularly suitable for further refinement by VNS and Path Relinking. In contrast, the original enhancement mechanisms of the ITROA and DHTROA, such as chaotic initialization, opposition-based learning, Golden Sine updating, and dynamic hierarchical strategies, are more generic and were originally developed for continuous optimization settings. After adaptation to the PFSP, these mechanisms appear to be less effective than the problem-specific initialization of the Hybrid TROA. Therefore, the results suggest that for the PFSP, the combination of strong scheduling-oriented initialization with effective local improvement is more beneficial than relying primarily on general-purpose exploration enhancement strategies.

### 4.5. Performance Evaluation Against Nature-Inspired Meta-Heuristics

We conducted computational experiments on benchmark datasets employing four prominent nature-inspired algorithms, namely the Hybrid GWO, Hybrid BA, Hybrid PSO, Hybrid ABC, Hybrid WOA, Hybrid TSO, Hybrid SSA, and Hybrid FA. Each algorithm was applied within the same framework as the Hybrid TROA. For each instance, we ran every method ten times and recorded the lowest value. The population of solutions for each meta-heuristic was set to 100, and the maximum number of iterations was set to 20. Table 5 presents the average mean error (AME) for each instance group for the makespan criterion. The Hybrid TROA consistently outperforms all other methods, except in the 20 × 10 instance set. The Hybrid SSA closely follows (0.98), while the Hybrid WOA ranks third (0.99), followed by the Hybrid GWO (1.00), together forming a group of strong competitors. The Hybrid FA and Hybrid TSO exhibit moderate performance across the instance sets, whereas the Hybrid BA, Hybrid PSO, and Hybrid ABC are less effective.

Table 6 presents the average mean error (AME) for each instance group under the TFT criterion. Overall, the Hybrid TROA achieves the best performance, with the lowest AME of 0.79 value. The Hybrid GWO is in second place with an AME of 0.82 value, while the Hybrid FA ranks third, and the Hybrid WOA ranks forth. Hybrid TSO (0.92) and the Hybrid SSA (0.97) also show competitive behavior, although they remain slightly inferior overall. In contrast, Hybrid PSO (1.20), Hybrid ABC (1.48), and especially the Hybrid BA (1.67) exhibit clearly weaker performance.

### 4.6. Performance Evaluation and Statistical Analysis of Optimization Methods for the Makespan Criterion

#### 4.6.1. Visual Analysis of Methods Performance Using Box Plots

**Figure 9:**
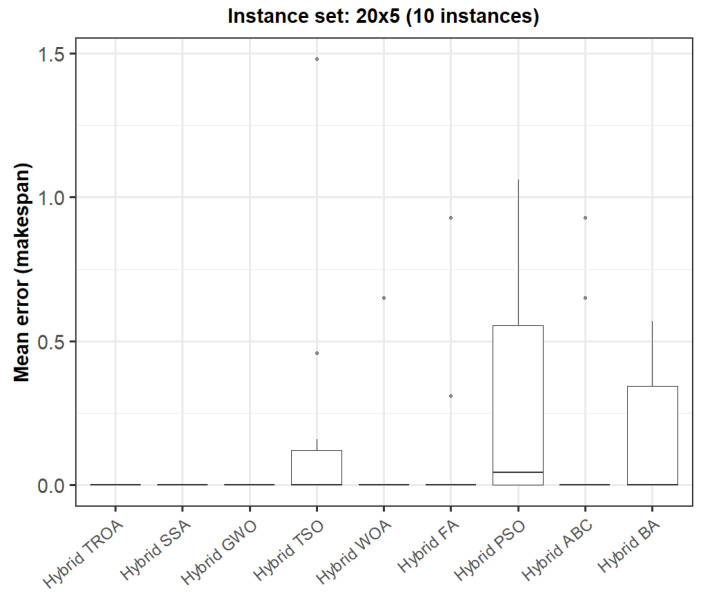
Box plot of the nine methods for instances 1 to 10 based on the makespan criterion. The dots represent outliers, reflecting deviations in performance and stability among the compared methods.

**Figure 10:**
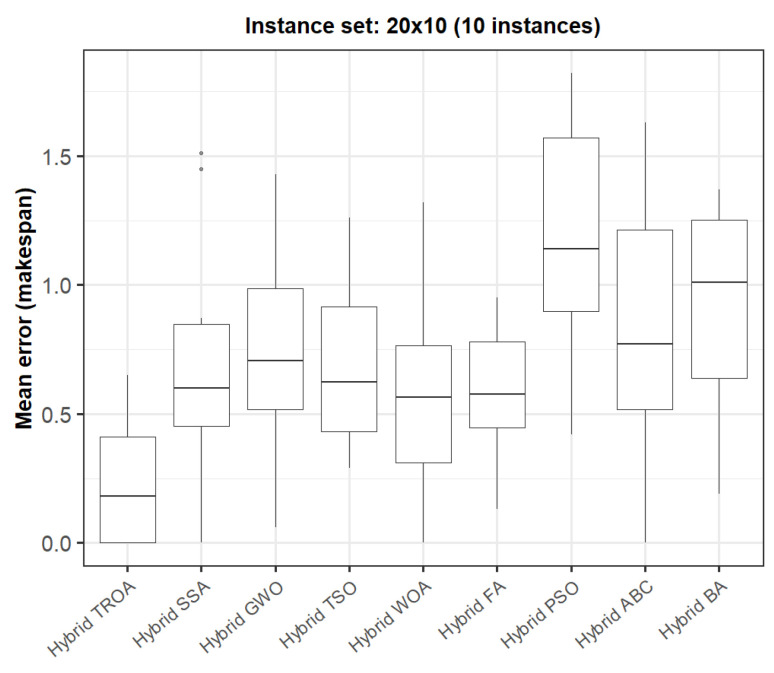
Box plot of the nine methods for instances 11 to 20 based on the makespan criterion. The dots represent outliers, reflecting deviations in performance and stability among the compared methods.

**Figure 11:**
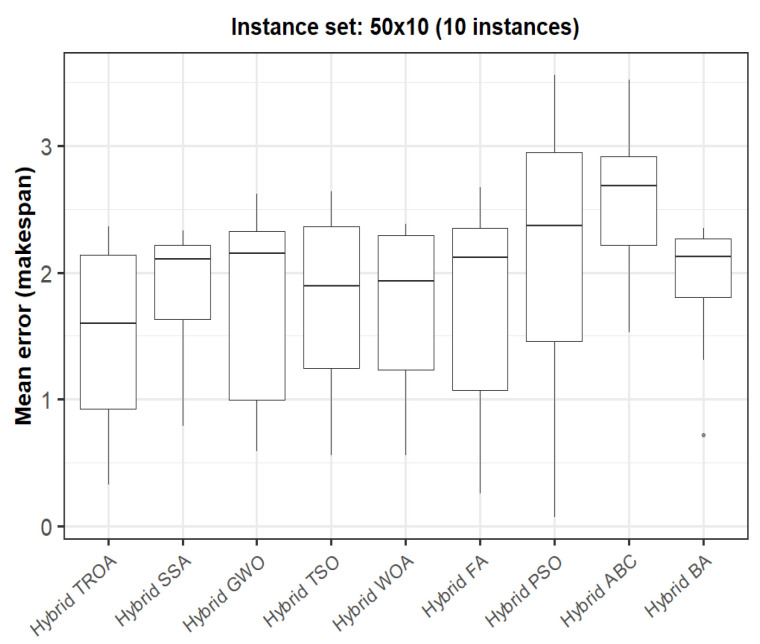
Box plot of the nine methods for instances 41 to 50 based on the makespan criterion. The dots represent outliers, reflecting deviations in performance and stability among the compared methods.

**Figure 12:**
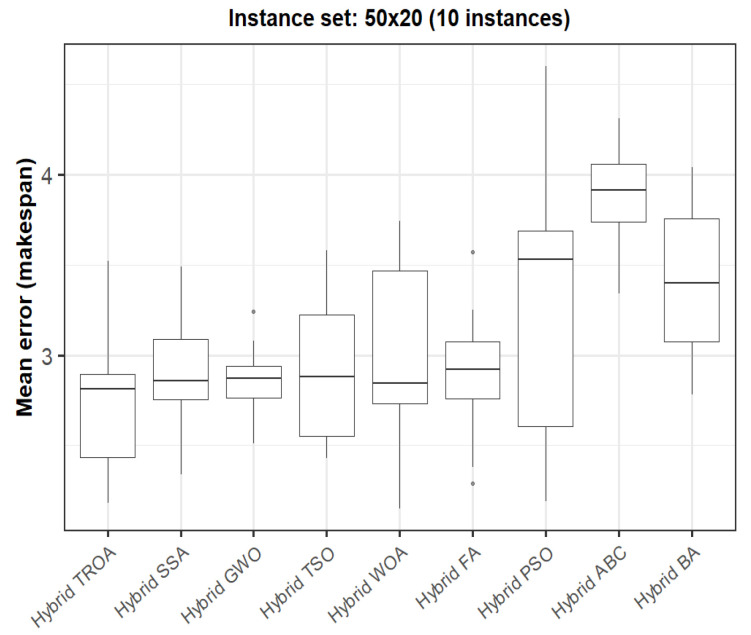
Box plot of the nine methods for instances 51 to 60 based on the makespan criterion. The dots represent outliers, reflecting deviations in performance and stability among the compared methods.

**Figure 13:**
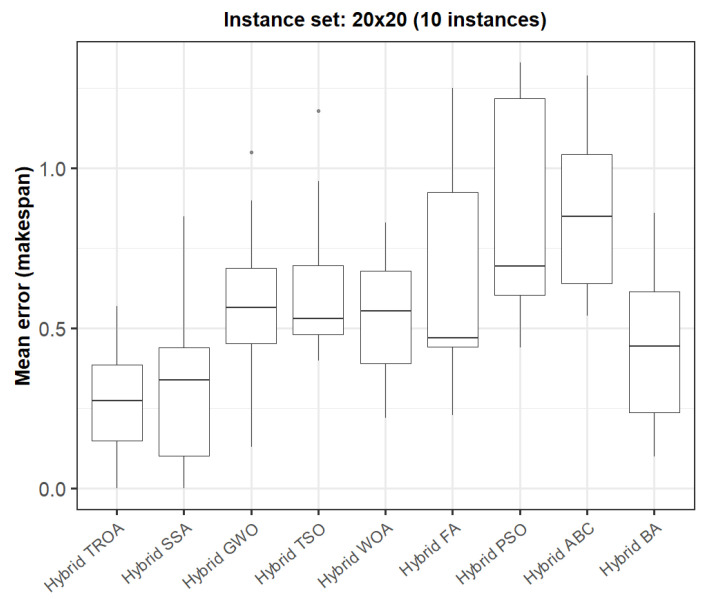
Box plot of the nine methods for instances 21 to 30 based on the makespan criterion. The dots represent outliers, reflecting deviations in performance and stability among the compared methods.

**Figure 14:**
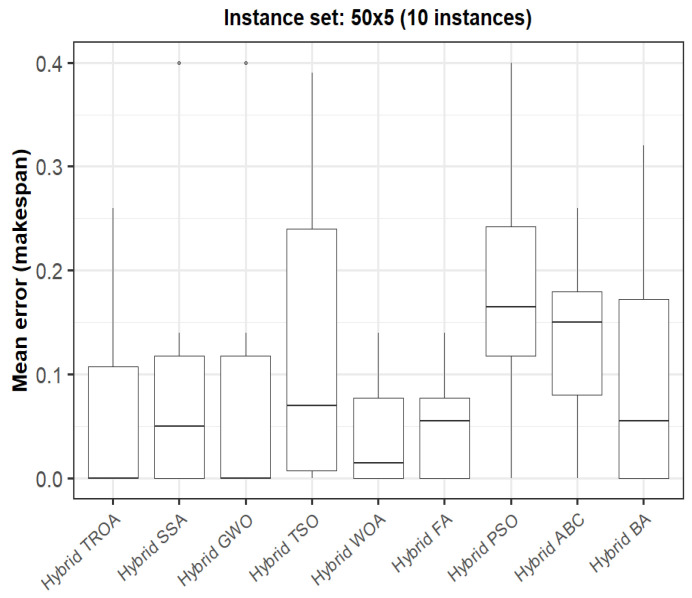
Box plot of the nine methods for instances 31 to 40 based on the makespan criterion. The dots represent outliers, reflecting deviations in performance and stability among the compared methods.

The box plots presented in Figure 9, Figure 10, Figure 11, Figure 12, Figure 13 and Figure 14 compare the distribution of the mean error values under the makespan criterion for nine methods, namely the Hybrid TROA, Hybrid PSO, Hybrid ABC, Hybrid BA, Hybrid GWO, Hybrid SSA, Hybrid FA, Hybrid TSO, and Hybrid WOA, across the 60 Taillard benchmark instances. For each instance and each method, the plotted value corresponds to the average result obtained over ten independent runs.

In the first group of instances (20 × 5, Figure 9), several methods, including the Hybrid TROA, Hybrid GWO, Hybrid SSA, and Hybrid WOA, attain median values equal or very close to zero, indicating highly competitive performance. However, Hybrid PSO and Hybrid TSO exhibit greater dispersion, while Hybrid ABC and the Hybrid FA also show a few larger error values, suggesting comparatively lower stability.

In the second group (20 × 10, Figure 10), the Hybrid TROA clearly presents the lowest median and one of the most compact distributions, whereas Hybrid PSO, the Hybrid BA, and Hybrid ABC show larger median errors and wider spreads.

In the third group (20 × 20, Figure 11), the Hybrid TROA again yields the lowest median value, followed by the Hybrid SSA and Hybrid BA, while Hybrid PSO and Hybrid ABC display the least favorable distributions.

In the fourth group (50 × 5, Figure 12), all methods achieve very low error values overall, although the Hybrid TROA and Hybrid GWO remain among the most stable and best-performing approaches.

In the fifth group (50 × 10, Figure 13), the superiority of the Hybrid TROA becomes more evident, as it attains the lowest median value, while Hybrid PSO and especially Hybrid ABC present substantially larger errors and higher variability.

Finally, in the sixth group (50 × 20, Figure 14), the Hybrid TROA continues to achieve the lowest median error, with the Hybrid GWO, Hybrid SSA, Hybrid WOA, Hybrid TSO, and Hybrid FA forming a closely competitive second tier, whereas the Hybrid BA, Hybrid PSO, and Hybrid ABC remain clearly weaker.

Considering all 60 instances together, the Hybrid TROA exhibits the lowest overall median error and a consistently favorable distribution, indicating both strong effectiveness and stability. The box plots also suggest that the Hybrid SSA, Hybrid FA, Hybrid WOA, Hybrid TSO, and Hybrid GWO form a competitive group with relatively similar overall behavior, although all remain slightly inferior to the Hybrid TROA. By contrast, Hybrid PSO and Hybrid ABC tend to produce larger errors and greater variability, especially on the larger and more difficult instance sets. Overall, the box-plot analysis supports the conclusion that the Hybrid TROA is the most effective and stable algorithm among the compared methods for minimizing the makespan in the PFSP.

#### 4.6.2. Non-Parametric Tests and *p*-Value Adjustments for Performance Comparison for the Makespan Criterion

The box plots offer an insight on the distribution, variability, and stability of the optimization methods. Additionally, they help us visualize which method has the lowest median objective value (better performance in minimization problems), how consistent the results are (smaller interquartile range indicates stability), and whether there are outliers that suggest performance fluctuations. From the other hand, non-parametric tests such as Friedman’s test, Friedman’s Aligned test, and the Quade test, determine whether there are statistically significant differences in performance among the methods. The lower the *p*-values, the stronger the hypothesis that the difference in performance is due to effectiveness and not chance.

Table 7 presents the average rankings, the F-statistics, and the corresponding p-values obtained from Friedman’s test, the Aligned Friedman test, and the Quade test for all compared methods. According to all three ranking schemes, the Hybrid TROA consistently attains the lowest rank values, indicating the best overall performance among the tested algorithms. More specifically, based on Friedman’s ranking, the Hybrid WOA is placed second, followed by the Hybrid SSA, while the Hybrid GWO and Hybrid FA follow closely. In contrast, Hybrid PSO and Hybrid ABC occupy the last positions, indicating the weakest overall behavior. Furthermore, the omnibus statistical results reveal highly significant differences among the compared methods, since the p-values of all three tests are extremely small. The corresponding F-statistics are also notably high, further supporting the conclusion that the observed performance differences are statistically significant and not due to random variation. Therefore, the results of Table 7 provide strong evidence that the Hybrid TROA is the most competitive and reliable method among the examined algorithms for solving the PFSP under the makespan criterion.

Comparisons with p-adjusted values help maintain the integrity of statistical conclusions when performing multiple tests. Bonferroni and Sidak are conservative, reducing Type I errors, while FDR methods allow for more findings at the risk of some false positives, which is ideal for exploratory research. Holm and Hommel offer balanced approaches.

The post hoc adjusted *p*-values following Friedman’s test, Friedman’s Aligned test, and Quade’s test (Table 8, Table A1 and Table A2 in Appendix A) consistently show that all pairwise comparisons between the Hybrid TROA and the remaining methods are statistically significant, since the adjusted *p*-values remain below the 0.05 significance level in all cases. In particular, the Hybrid TROA exhibits very strong statistical superiority over Hybrid ABC, Hybrid PSO, the Hybrid BA, and Hybrid TSO, as their adjusted *p*-values are extremely small, even after multiple-testing corrections. Statistically significant differences are also observed against the Hybrid GWO and Hybrid FA. The highest adjusted *p*-values are obtained in the comparisons with the Hybrid WOA and Hybrid SSA, indicating that these two methods are the closest competitors to the Hybrid TROA, although the Hybrid TROA still performs significantly better overall.

Therefore, the post hoc analysis confirms that the Hybrid TROA is the best-performing method for solving the PFSP with the makespan criterion among the compared algorithms, while the Hybrid WOA and Hybrid SSA emerge as the strongest alternative approaches, and Hybrid PSO and Hybrid ABC exhibit the weakest overall performance.

### 4.7. Performance Evaluation and Statistical Analysis of Optimization Methods for the Total Flow Time Criterion

#### 4.7.1. Visual Analysis of Methods Performance Using Box Plots for the Total Flow Time Criterion

**Figure 15:**
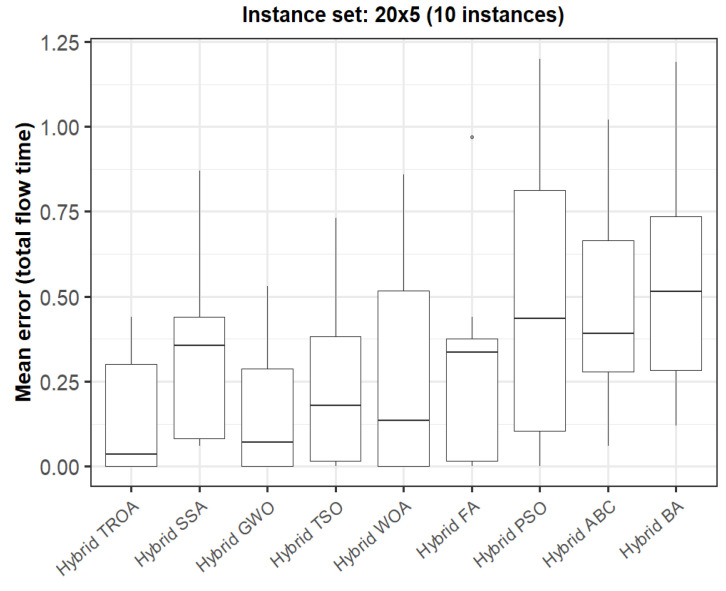
Box plot of the nine methods for instances 1 to 10 based on the total flow time criterion. The dots represent outliers, reflecting deviations in performance and stability among the compared methods.

**Figure 16:**
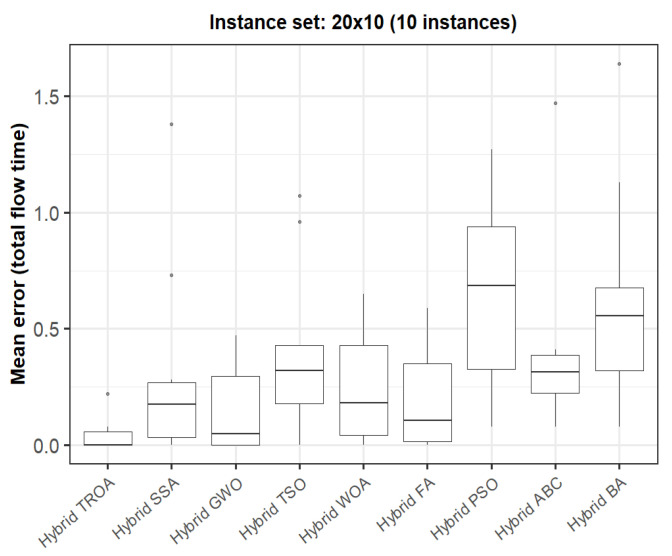
Box plot of the nine methods for instances 11 to 20 based on the total flow time criterion. The dots represent outliers, reflecting deviations in performance and stability among the compared methods.

**Figure 17:**
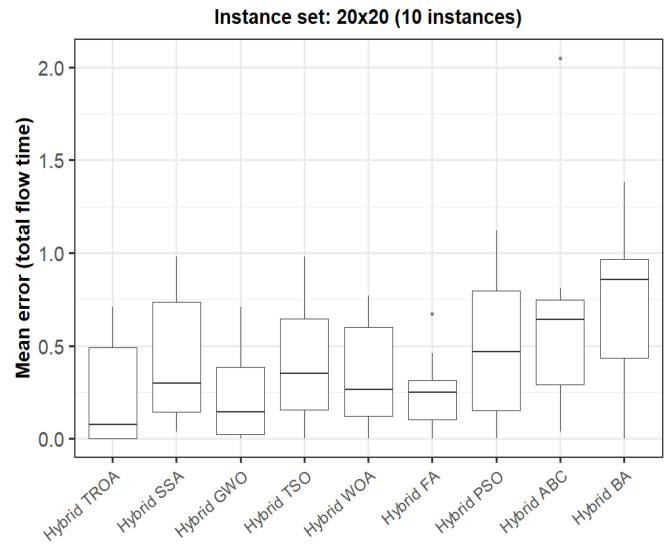
Box plot of the nine methods for instances 21 to 30 based on the total flow time criterion. The dots represent outliers, reflecting deviations in performance and stability among the compared methods.

**Figure 18:**
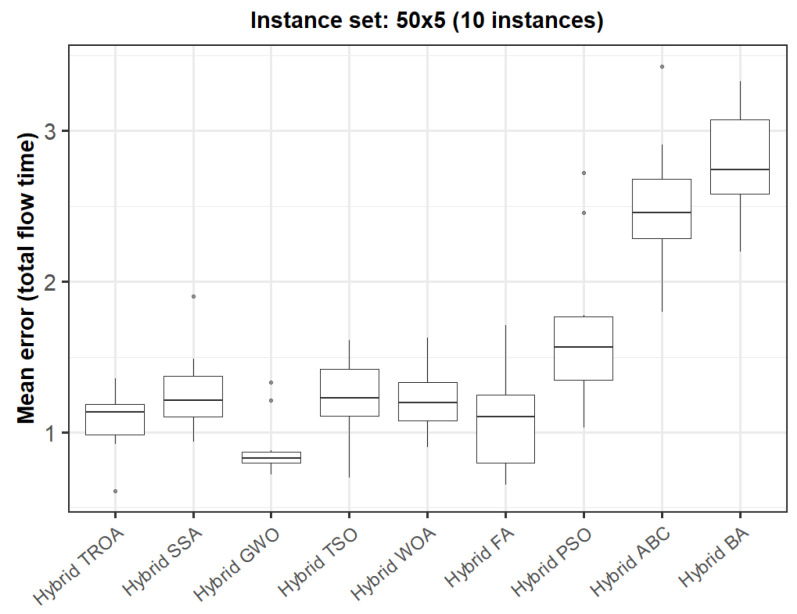
Box plot of the nine methods for instances 31 to 40 based on the total flow time criterion. The dots represent outliers, reflecting deviations in performance and stability among the compared methods.

**Figure 19:**
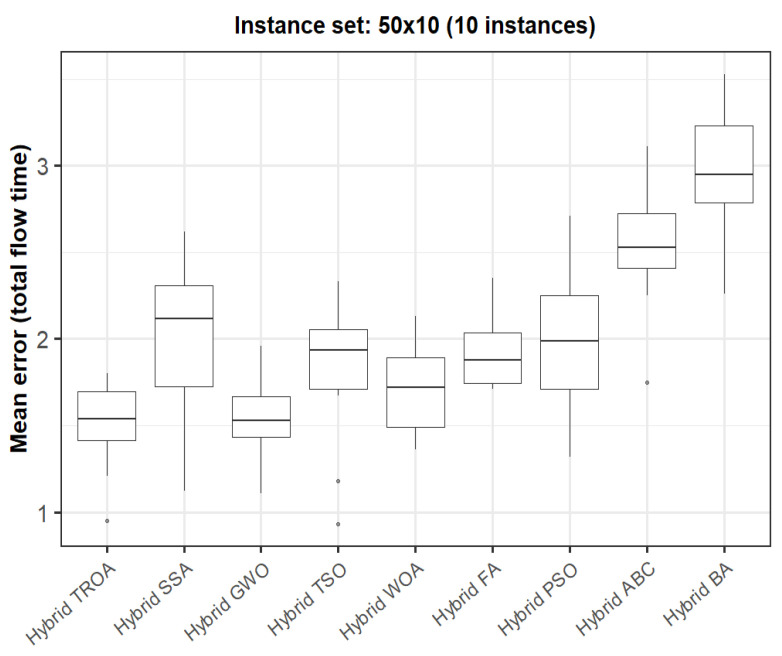
Box plot of the nine methods for instances 41 to 50 based on the total flow time criterion. The dots represent outliers, reflecting deviations in performance and stability among the compared methods.

**Figure 20:**
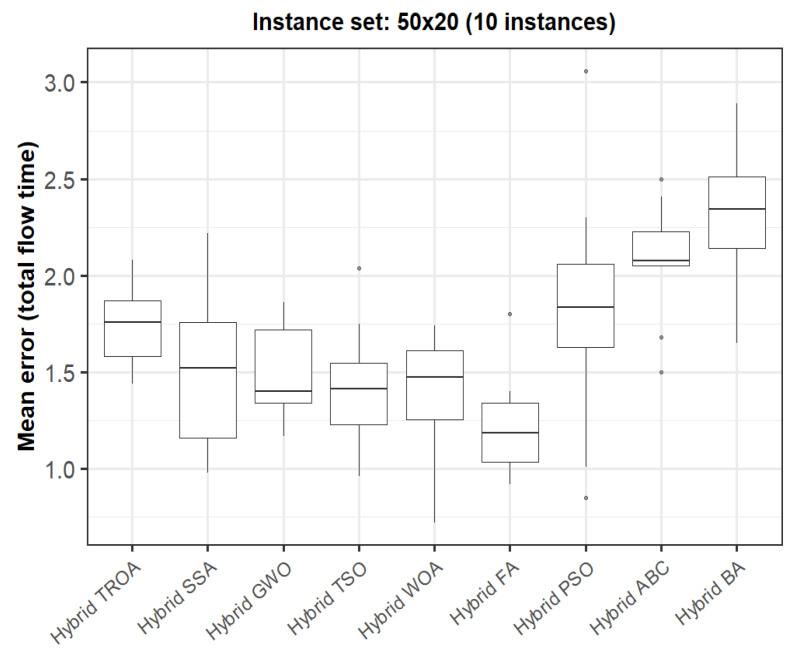
Box plot of the nine methods for instances 51 to 60 based on the total flow time criterion. The dots represent outliers, reflecting deviations in performance and stability among the compared methods.

The box plots in Figure 15, Figure 16, Figure 17, Figure 18, Figure 19 and Figure 20 compare the distribution of the mean error values under the total flow time (TFT) criterion for nine methods, namely the Hybrid TROA, Hybrid GWO, Hybrid PSO, Hybrid ABC, Hybrid BA, Hybrid SSA, Hybrid FA, Hybrid TSO, and Hybrid WOA, over the 60 Taillard benchmark instances. For each instance and each method, the plotted value corresponds to the average result obtained over ten independent runs.

For the first instance set (20 × 5, Figure 15), the Hybrid TROA and Hybrid GWO exhibit the lowest median values, indicating the best overall performance in this group. The Hybrid WOA and Hybrid TSO also remain competitive, whereas Hybrid PSO, Hybrid ABC, and the Hybrid BA show higher medians and wider spreads, suggesting weaker and less stable behavior. The Hybrid SSA and Hybrid FA occupy an intermediate position.

In the second instance set (20 × 10, Figure 16), the Hybrid TROA clearly achieves the lowest median and one of the narrowest spreads, indicating both strong effectiveness and stability. The Hybrid GWO and Hybrid FA follow, while Hybrid PSO and the Hybrid BA display considerably larger error values. The box plots therefore indicate a clear advantage for the Hybrid TROA in this instance group.

For the third instance set (20 × 20, Figure 17), the Hybrid TROA again attains the lowest median value, followed by the Hybrid GWO, Hybrid FA, and Hybrid WOA. In contrast, the Hybrid BA, Hybrid ABC, and Hybrid PSO exhibit noticeably higher medians and broader dispersion. Thus, in this group, the Hybrid TROA remains the most favorable method, with the Hybrid GWO constituting its closest competitor.

In the fourth instance set (50 × 5, Figure 18), the performance profile changes slightly, as the Hybrid GWO achieves the lowest median value, while the Hybrid TROA follows very closely. The Hybrid FA, Hybrid WOA, Hybrid SSA, and Hybrid TSO form a competitive intermediate group, whereas Hybrid ABC and especially the Hybrid BA yield substantially higher errors. Therefore, although the Hybrid TROA remains highly competitive, the Hybrid GWO appears slightly stronger for this group.

For the fifth instance set (50 × 10, Figure 19), the Hybrid TROA once again provides the best median performance, followed closely by the Hybrid GWO. The Hybrid WOA remains competitive, while the Hybrid FA, Hybrid TSO, Hybrid PSO, and Hybrid SSA exhibit somewhat larger medians. Hybrid ABC and the Hybrid BA continue to show the weakest behavior. Hence, the Hybrid TROA regains the leading position in this more difficult group of instances.

Finally, in the sixth instance set (50 × 20, Figure 20), the Hybrid FA achieves the lowest median value, while the Hybrid GWO, Hybrid TSO, Hybrid WOA, and Hybrid TROA form a closely competitive group. Although the Hybrid TROA is no longer the best-performing method in this last group, it still remains among the strongest methods overall. By contrast, Hybrid PSO, Hybrid ABC, and the Hybrid BA again produce larger error values, indicating inferior performance.

Across the 60 instances, the Hybrid TROA and Hybrid GWO emerge as the most stable and effective methods for minimizing the total flow time, consistently producing lower objective values with minimal fluctuations. Of the two, the Hybrid TROA stands out as the better option, as it consistently delivers slightly lower objective values for the total flow time criterion across the majority of instances.

#### 4.7.2. Non-Parametric Tests and *p*-Value Adjustments for Performance Comparison for the Total Flow Time Criterion

The statistical comparisons between the Hybrid TROA and other methods using multiple correction techniques (Bonferroni, Holm, FDR-BY, SIDAK, FDR-BH, and Hommel) reveal key insights into performance differences. The non-parametric analysis in Table 9 confirms that statistically significant differences exist among the compared methods, as indicated by the Friedman, Aligned Friedman, and Quade tests.

The post hoc comparisons (Table 10, Table A3 and Table A4 in the Appendix A) further show that the Hybrid TROA significantly outperforms Hybrid PSO, Hybrid ABC, the Hybrid BA, the Hybrid SSA, and Hybrid TSO, since the adjusted *p*-values remain very small under all correction procedures. By contrast, the comparisons between the Hybrid TROA and Hybrid GWO yield high adjusted *p*-values in all three post hoc analyses, indicating that these two methods are statistically comparable.

The comparisons with the Hybrid WOA and Hybrid FA are less conclusive, as significance is observed in some cases but is not confirmed consistently by the Aligned Friedman and Quade procedures. Therefore, these two methods may also be regarded as competitive alternatives, although they remain inferior to the Hybrid TROA in the overall ranking.

Overall, the results identify the Hybrid TROA as the best-ranked method for the TFT criterion, with the Hybrid GWO emerging as its closest competitor.

### 4.8. Comparisons Against Other Nature-Inspired Algorithms from the Literature with the Makespan Criterion

The proposed hybridization scheme appears to be more efficient when combined with the TROA than with the SSA, GWO, TSO, WOA, FA, PSO, ABC, and BA. To further establish the effectiveness of the proposed Hybrid TROA, we compared its performance in terms of the AME with the makespan criterion with that of other seven nature-inspired meta-heuristics: Q-learning Artificial Bee Colony (QABC) [29], Chaos Whale Algorithm (CWA) [30], Hybrid Adaptive Particle Swarm Optimization (HAPSO) [31], Improved Artificial Bee Colony (IABC) [32], Hybrid Monkey Search Algorithm (HMSA) [33], Discrete Differential Evolution (DDE) [34], and Particle Swarm Optimization with Expanding Neighborhood Topology (PSOENT) [35]. These meta-heuristics were selected because they are competitive population-based, nature-inspired methods reported in the PFSP for the makespan in the literature. Several of them incorporate hybrid improvement mechanisms similar to those used in the proposed approach. For instance, NEH is employed for population initialization in IABC, DDE and the CWA, whereas VNS and Path Relinking are incorporated in HAPSO and PSOENT. Other local search schemes are also used in the HMSA, IABC, DDE, and CWA.

The results in Table 11 demonstrate that the Hybrid TROA is one of the most competitive algorithms among the compared methods. Although it does not achieve the best performance in all instance groups, it obtains a low overall AME and outperforms several established literature-based meta-heuristics.

### 4.9. Limitations and Applicability of the Proposed Method

Despite the strong empirical performance of the Hybrid TROA on the examined PFSP instances, several limitations should be acknowledged. First, the computational cost of the method is relatively high due to the hybridization strategy and the integration of local search mechanisms operating on permutation-based solution spaces. This becomes more pronounced as the problem size increases, particularly for large-scale instances from well-known benchmark sets such as Taillard, where the factorial growth of the search space significantly impacts computational effort.

Second, the performance of the algorithm is sensitive to parameter settings that regulate the balance between exploration and exploitation. Although appropriate parameter values were determined experimentally, different PFSP instance characteristics (e.g., job-to-machine ratio, processing time distributions, or degree of instance heterogeneity) may require additional tuning for optimal performance.

Furthermore, as with most meta-heuristic approaches for the PFSP, there is no guarantee of global optimality, and the algorithm’s effectiveness may vary depending on the structure of the permutation landscape, particularly in instances with high ruggedness or strong local optima.

Nevertheless, the proposed Hybrid TROA is particularly suitable for medium- to large-scale PFSP instances where obtaining high-quality solutions is more critical than achieving exact optimality within limited computational time.

## 5. Balance Optimization for Energy-Efficient Scheduling in the Permutation Flowshop Problem

### 5.1. The Energy-Efficient PFSP

The fundamental assumptions for the Energy-Efficient PFSP are inspired by the studies of Fang et al. [36] and Öztop et al. [37] and an extension of our previous study [38].

The Processing Time is Inversely Proportional to Speed Raised to Power of β and Proportional to an Efficient Factor:

If the processing time at speed 1 is $p_{ij}$ for job i on machine j, then at speed s, the processing time $p_{ij}(s)$ is:

(18) $$p_{ij}(s)=\frac{p_{ij}(1)}{s^{\beta }}\cdot \eta (s)$$

where s∈{1,2,3} is the speed factor (unitless), $p_{ij}(1)$ is the processing time at speed 1, β is a constant that controls the effect of speed on processing time, within the range [1, 2] (in our study is set equal to 0.9), and η(s) is an efficient factor that adjusts the processing time to account for additional inefficiencies at higher speeds and is calculated as follows:

(19) $$\eta \left(s\right)=1+\gamma (s-1)$$

where γ is a constant representing how inefficiency increases with speed beyond the base level (s = 1), accounting for factors like friction, wear, and cooling demands.

Speed factor s is a unitless multiplier of the base rotational speed (RPM1), where s = 1 equals RPM1, s = 2 equals 2 × RPM1, and so on.

The Energy Consumption is Based on the Processing Time: The energy consumption at speed 1 is equal to the processing time:

(20) $$E_{ij}(1)=p_{ij}(1)$$

The Power Consumption Model:

Power consumption is assumed to follow a power function of speed, as presented in [18]:

(21) $$P(s)=P_{0}\cdot s^{a}$$

where a is an exponent (set to 3) that defines how power scales with speed.

$P_{0}$ is the power at minimum speed (s = 1), set to 1.5 kW, reflecting typical low-speed consumption of small CNC machines (0.5–5 kW). P(s), also in kW, represents power at speed s.

Total Energy Consumption Relationship:

Energy consumption for job i on machine j at speed s is:

(22) $$E_{ij}\left(s\right)=P\left(s\right)\cdot p_{ij}(s)$$

Since P(s) is measured in kW and $p_{ij}(s)$ in time units (for simplicity, we assume this is measured in seconds), then the energy consumption is measured in kJ.

Substituting previous assumptions:

(23) $$E_{ij}\left(s\right)=P_{0}\cdot s^{a}\cdot \frac{p_{ij}\left(1\right)}{s^{\beta }}\eta (s)=P_{0}\cdot s^{a-\beta }\cdot \eta (s)\cdot p_{ij}(1)$$

The total energy consumption is calculated as follows:

(24) $$E_{total}\left(s\right)=\sum_{i=1}^{n}\sum_{j=1}^{m}E_{ij}(s)$$

### 5.2. Proposed Objective Function for Balancing Makespan and Energy Consumption

In the Energy-Efficient PFSP, minimizing makespan $C_{max}$ often leads to disproportionately high energy consumption (E) due to the power relationship between energy and speed. The study of Ding et al. [21] suggests that higher speeds lead to smaller processing times but increase energy consumption. To balance this trade-off, we proposed the following objective function:

(25) $$Z=\frac{w_{1}\cdot \frac{C_{max}}{C_{max}^{worst}}+w_{2}\cdot \frac{E}{E^{worst}}}{w_{1}+w_{2}}$$

where $w_{1}=\frac{E^{worst}}{C_{max}^{worst}}$, and $w_{2}=1.$

By setting $w_{1}$ as the ratio of worst-case energy to worst-case makespan, the makespan term is scaled to match the magnitude of energy consumption, preventing one factor from overshadowing the other. By setting $w_{2}\;$= 1, the natural scale of energy consumption is preserved. By dividing by $w_{1}+w_{2}$, we normalize the objective Z, ensuring that both the makespan and energy consumption contribute equally in a relative sense.

### 5.3. Computational Experiments on Benchmark Datasets

In order to conduct computational experiments, Taillard’s [17] benchmark datasets are utilized. The Hybrid TROA and Hybrid GWO were each run three times per instance, with the best outcome recorded. Table 12 presents four metrics: C_max_ (makespan), energy in kJ (total energy used), objective Z (as defined in Equation (25)), and total kWh, which reflects the average hourly energy consumption based on machine speed and schedule configuration. Total kWh is calculated as follows:

(26) $$Total\;kWh=\frac{Energy}{3600}$$

The metric %$\Delta C_{max}$ represents the percentage difference of $C_{max}^{GWO}$ and $C_{max}^{TROA}$ divided by $C_{max}^{GWO}$, as shown in the following equation:

(27) $$\%\Delta C_{max}=\frac{C_{max}^{GWO}-C_{max}^{TROA}}{C_{max}^{GWO}}\cdot 100$$

Negative values indicate that $C_{max}^{TROA}$ is greater than $C_{max}^{GWO}$, meaning that the GWO achieved a shorter makespan. Similarly, the %ΔΕ metric is calculated as follows:

(28) $$\%\Delta E=\frac{E^{GWO}-E^{TROA}}{E_{max}^{GWO}}\cdot 100$$

This equation represents the percentage difference in total energy consumption between the two algorithms.

Table 12 is divided into two sections: the first shows the results for the Hybrid TROA, and the second shows the results for the Hybrid GWO. For each section, the first column displays the makespan values, the second column reports energy consumption (kJ), the third column lists the objective function values, and the fourth column gives the total power consumption (kWh). The last two columns show the percentage difference between the Hybrid GWO and Hybrid TROA, one for the makespan and the other for energy consumption.

**Figure 21:**
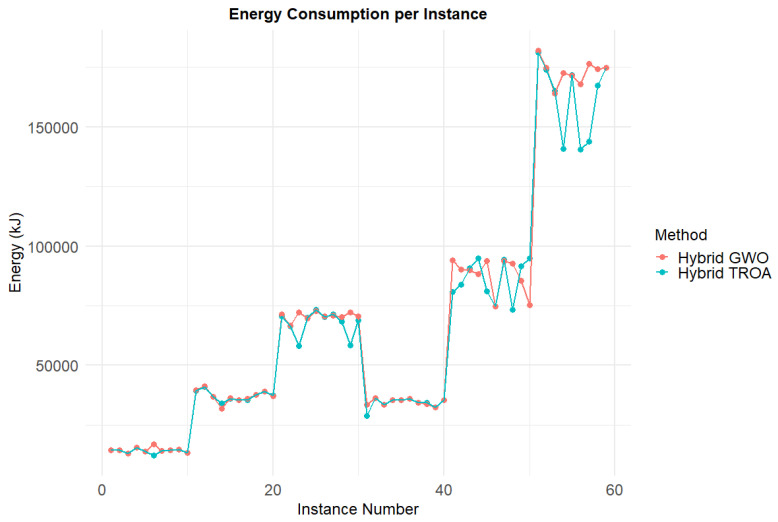
Comparative analysis of energy consumption for all methods across the complete set of instances.

**Figure 22:**
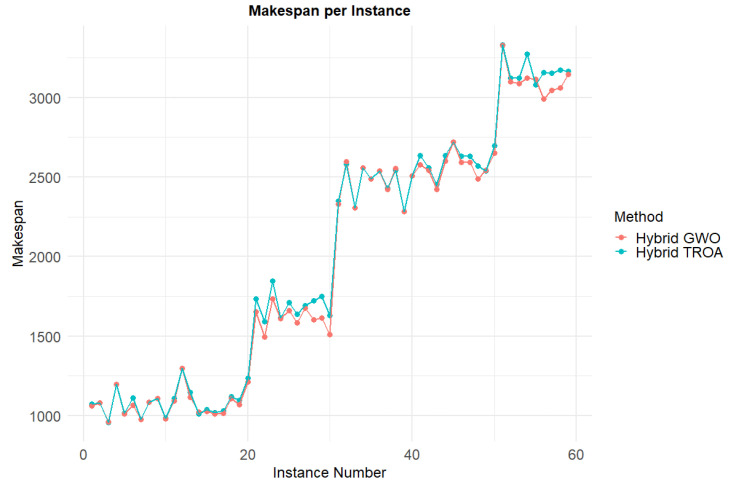
Comparative analysis of makespan performance for all methods across the complete set of instances.

**Figure 23:**
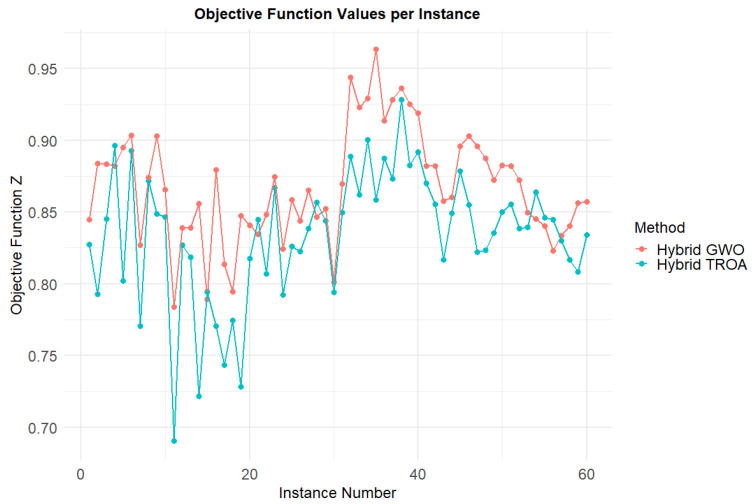
Comparison of objective function values among methods across all instances.

Figure 21 presents a visual comparison of the energy consumption for each instance between the two methods. In most cases, the Hybrid TROA exhibits lower energy consumption, while in the last two datasets, it outperforms the other method significantly. Figure 22 shows the comparison of makespan values for both methods across all instances. The Hybrid GWO more frequently achieves lower makespan values, and in the last dataset, it provides superior results compared to the other method. Figure 23 displays the objective function values for each instance and method. Lower values indicate more balanced solutions, meaning that the optimization process minimizes both targets effectively. The Hybrid TROA achieves better results for most instances, with only a few exceptions, and is therefore the most suitable method for tackling the Energy-Efficient PFSP.

## 6. Conclusions

In this study, we demonstrated the effectiveness of the Hybrid TROA in solving the Permutation Flowshop Scheduling Problem (PFSP) considering the makespan and the total flow time criteria by integrating it with Variable Neighborhood Search and Path Relinking strategies. In summary, the proposed Hybrid TROA demonstrated strong and consistent performance across all experimental comparisons. First, its evaluation against partial hybrid variants, namely TROA-VNS-PR, TROA-NEH-PR, and the classical TROA, showed that the simultaneous integration of all hybridization components is more effective than their partial application. In addition, the Hybrid TROA outperformed other TROA-based variants, such as the ITROA and DHTROA, when adapted to the PFSP. Finally, the proposed method also proved superior to eight well-established meta-heuristics—the GWO, PSO, BA, ABC, TSO, WOA, SSA, and FA—over the benchmark instances examined. The statistical analysis further supported these findings, confirming the robustness and effectiveness of the Hybrid TROA for the PFSP under both the makespan and total flow time criteria.

The comparisons between the Hybrid TROA and several population-based meta-heuristics from the literature highlight the consistently strong and competitive performance of the proposed method, which achieves better average results than most of the compared methods, thus confirming the effectiveness of the proposed hybridization scheme.

Furthermore, this study proposed an objective function for the Energy-Efficient PFSP that jointly considers makespan and energy consumption. The experimental results with the Hybrid GWO and Hybrid TROA provided useful insights, showing that the Hybrid GWO was more effective in reducing the makespan, while the Hybrid TROA was more efficient in minimizing energy consumption. Overall, the Hybrid TROA achieved lower objective function values, suggesting that it offers a more balanced and effective optimization approach for the EE-PFSP.

The results highlight the TROA as a reliable and effective meta-heuristic for industrial scheduling problems where productivity and energy efficiency must be considered concurrently. Future research may focus on integrating the proposed method into multi-objective optimization frameworks, as well as extending its application to larger-scale and more complex problem instances.

## Figures and Tables

**Table 1:** Results of Hybrid TROA for PFSP with makespan $\left(C_{max}\right)$ for Taillard’s benchmark datasets.

Instances			BKS	Hybrid TROA	AVG	St. Dev.	ME (%)	Instances			BKS	Hybrid TROA	AVG	St. Dev.	ME (%)
*N*	*n*	*m*	$C_{max}$	$C_{max}$	$C_{max}$			*N*	*n*	*m*	$C_{max}$	$C_{max}$	$C_{max}$		
1	20	5	1278	1278	1278	0.00	0.00	31	50	5	2724	2724	2728	2.11	0.00
2	20	5	1359	1359	1359.9	0.00	0.00	32	50	5	2834	2838	2841.1	4.77	0.14
3	20	5	1081	1081	1089.9	5.57	0.00	33	50	5	2621	2621	2624.4	2.07	0.00
4	20	5	1293	1293	1298.8	5.57	0.00	34	50	5	2751	2753	2761.5	5.58	0.40
5	20	5	1235	1235	1241.6	3.24	0.00	35	50	5	2863	2863	2863.9	0.32	0.03
6	20	5	1195	1195	1195.8	3.24	0.00	36	50	5	2829	2829	2830.9	1.10	0.07
7	20	5	1239	1239	1239	0.00	0.00	37	50	5	2725	2732	2732.3	0.95	0.11
8	20	5	1206	1206	1208.6	0.00	0.00	38	50	5	2683	2683	2685.2	2.04	0.00
9	20	5	1230	1230	1242.8	8.50	0.00	39	50	5	2552	2555	2558.2	3.01	0.12
10	20	5	1108	1108	1108	8.50	0.00	40	50	5	2782	2782	2782.1	0.32	0.00
AME							0.00								0.06
11	20	10	1582	1582	1595.4	6.29	0.00	41	50	10	2991	3054	3074.7	13.33	2.11
12	20	10	1659	1669	1680.7	9.65	0.60	42	50	10	2867	2931	2943.7	10.64	2.23
13	20	10	1496	1501	1512.1	9.04	0.33	43	50	10	2839	2906	2924.3	9.91	2.36
14	20	10	1377	1378	1391.9	5.65	0.07	44	50	10	3063	3087	3104.7	9.35	0.78
15	20	10	1419	1419	1431.5	8.13	0.00	45	50	10	2976	3026	3061.6	18.54	1.68
16	20	10	1397	1401	1416.6	8.03	0.29	46	50	10	3006	3026	3075.1	19.89	0.67
17	20	10	1484	1484	1489.7	4.03	0.00	47	50	10	3093	3140	3158.2	11.87	1.52
18	20	10	1538	1548	1556.5	5.40	0.65	48	50	10	3037	3047	3067.3	15.43	0.33
19	20	10	1593	1593	1608.1	8.84	0.00	49	50	10	2897	2936	2953.3	13.90	1.35
20	20	10	1591	1598	1617.3	9.12	0.44	50	50	10	3065	3131	3154	13.18	2.15
AME							0.24								1.52
21	20	20	2297	2303	2319.1	9.15	0.26	51	50	20	3850	3961	3981	18.24	2.88
22	20	20	2099	2105	2118	7.04	0.29	52	50	20	3704	3790	3829	18.38	2.32
23	20	20	2326	2336	2352.7	12.48	0.43	53	50	20	3640	3768	3782.4	10.49	3.52
24	20	20	2223	2227	2237.5	5.78	0.18	54	50	20	3720	3824	3854.8	14.97	2.80
25	20	20	2291	2304	2315.7	6.90	0.57	55	50	20	3610	3710	3739.3	17.73	2.77
26	20	20	2226	2228	2245.5	9.68	0.09	56	50	20	3681	3765	3796.7	21.13	2.28
27	20	20	2273	2281	2293	7.09	0.35	57	50	20	3704	3818	3840.7	16.01	3.08
28	20	20	2200	2200	2217.4	8.75	0.00	58	50	20	3691	3798	3831.8	25.87	2.90
29	20	20	2237	2246	2258.9	6.24	0.40	59	50	20	3743	3849	3871.4	18.49	2.83
30	20	20	2178	2181	2199.3	11.11	0.14	60	50	20	3756	3838	3876.4	18.45	2.18
AME							0.27								2.76

**Table 2:** Results of the Hybrid TROA for the PFSP with the total flow time (TFT) for Taillard’s benchmark datasets.

Instances			BKS	Hybrid TROA	AVG	St. Dev.	ME (%)	Instances			BKS	Hybrid TROA	AVG	St. Dev.	ME (%)
*N*	*n*	*m*	TFT	TFT	TFT			*N*	*n*	*m*	TFT	TFT	TFT		
1	20	5	14,033	14,033	14,043.4	7.84	0.00	31	50	5	64,838	65,608	66,066.6	208.31	1.19
2	20	5	15,151	15,217	15,275	51.50	0.44	32	50	5	68,202	68,949	69,389.6	216.20	1.10
3	20	5	13,301	13,301	13,342	31.45	0.00	33	50	5	63,436	64,179	64,646.2	289.24	1.17
4	20	5	15,447	15,484	15,547	47.50	0.24	34	50	5	68,536	69,165	69,635.2	314.20	0.92
5	20	5	13,529	13,529	13,572.4	29.48	0.00	35	50	5	69,496	70,181	70,410.6	140.84	0.99
6	20	5	13,123	13,123	13,151.7	22.98	0.00	36	50	5	67,034	67,444	67,850.4	267.35	0.61
7	20	5	13,548	13,558	13,602.3	33.08	0.07	37	50	5	66,338	67,220	67,390.9	130.18	1.33
8	20	5	13,948	13,948	13,986.7	29.46	0.00	38	50	5	64,521	65,154	65,467.7	292.86	0.98
9	20	5	14,295	14,346	14,390.5	21.48	0.36	39	50	5	63,103	63,847	64,157.9	172.50	1.18
10	20	5	12,943	12,984	13,015.5	22.12	0.32	40	50	5	69,121	70,058	70,334.6	155.63	1.36
AME							0.14								1.08
11	20	10	20,911	20,911	20,988.2	56.51	0.00	41	50	10	87,548	88,780	89,645.3	357.40	1.41
12	20	10	22,440	22,440	22,656.2	144.81	0.00	42	50	10	83,115	84,516	84,822.1	288.01	1.69
13	20	10	19,833	19,833	19,906.8	43.40	0.00	43	50	10	80,275	81,426	81,716.5	261.32	1.43
14	20	10	18,710	18,751	18,790.7	30.84	0.22	44	50	10	86,856	87,684	88,288.9	280.23	0.95
15	20	10	18,641	18,644	18,723.8	52.41	0.02	45	50	10	86,661	87,943	88,422.2	339.65	1.48
16	20	10	19,245	19,245	19,383.9	75.37	0.00	46	50	10	86,735	88,124	88,659.1	310.57	1.60
17	20	10	18,363	18,376	18,396.6	29.96	0.07	47	50	10	89,014	90,614	90,853.2	163.86	1.80
18	20	10	20,241	20,241	20,277.6	46.12	0.00	48	50	10	87,192	88,676	89,099	352.36	1.70
19	20	10	20,330	20,330	20,387.1	54.58	0.00	49	50	10	85,884	86,927	87,705.7	439.23	1.21
20	20	10	21,320	21,337	21,348.6	13.28	0.08	50	50	10	88,149	89,733	90,474.9	377.20	1.80
AME							0.04								1.51
21	20	20	33,623	33,812	33,896.6	69.88	0.56	51	50	20	126,118	127,859	128,320.1	266.50	1.75
22	20	20	31,587	31,604	31,778.6	105.08	0.05	52	50	20	119,513	120,467	121,350.8	537.30	1.54
23	20	20	33,920	33,920	33,977.3	42.76	0.00	53	50	20	116,916	117,894	118,648	549.10	1.48
24	20	20	31,661	31,661	31,706.4	25.18	0.00	54	50	20	121,203	123,032	123,483.8	355.64	1.88
25	20	20	34,557	34,590	34,678.8	73.32	0.10	55	50	20	118,783	120,325	120,969.3	373.72	1.84
26	20	20	32,564	32,564	32,776.5	159.79	0.00	56	50	20	120,914	122,540	123,423.2	331.37	2.08
27	20	20	32,922	33,142	33,188.7	24.76	0.67	57	50	20	123,583	125,403	125,695.6	172.90	1.71
28	20	20	32,412	32,412	32,497.6	41.43	0.00	58	50	20	122,900	124,276	125,071.8	386.32	1.77
29	20	20	33,600	33,693	33,761.1	79.12	0.28	59	50	20	122,147	124,130	124,481.7	240.23	1.91
30	20	20	32,262	32,491	32,543.1	48.51	0.71	60	50	20	124,529	125,744	126,322.5	334.64	1.44
AME							0.24								1.74

**Table 3:** Comparisons of Hybrid TROA and three other TROA variations.

Instance	Hybrid TROA	TROA	TROA-NEH-PR	TROA-VNS-PR
Ta001	0.00	0.00	0.00	0.00
Ta011	0.00	0.70	1.01	1.14
Ta021	0.26	0.91	0.44	1.48
Ta031	0.00	0.18	0.18	0.18
Ta041	2.11	3.24	3.08	3.28
Ta051	2.88	3.77	3.51	4.02
AVG	0.88	1.47	1.37	1.68

**Table 4:** Comparisons of Hybrid TROA against ITROA and DHTROA.

Instance	Hybrid TROA	ITROA	DHTROA
Ta001	0.00	0.08	0.00
Ta011	0.00	0.19	1.83
Ta021	0.26	0.04	1.39
Ta031	0.00	0.00	0.18
Ta041	2.11	5.15	6.49
Ta051	2.88	5.25	6.52
AVG	0.88	1.78	2.74

**Table 5:** The average mean errors for the makespan criterion of the nine methods for each instance set.

Instances’ Group	Hybrid TROA	Hybrid GWO	Hybrid BA	Hybrid PSO	Hybrid ABC	Hybrid WOA	Hybrid TSO	Hybrid SSA	Hybrid FA
10 × 5	0.00	0.00	0.16	0.29	0.16	0.06	0.23	0.00	0.12
10 × 10	0.24	0.73	0.90	1.19	0.85	0.58	0.63	0.69	0.59
20 × 5	0.27	0.57	0.44	0.85	0.87	0.53	0.64	0.34	0.63
20 × 10	0.06	0.08	0.09	0.18	0.13	0.04	0.15	0.09	0.05
50 × 10	1.52	1.74	1.92	2.14	2.57	1.72	1.70	1.87	1.77
50 × 20	2.76	2.88	3.42	3.28	3.86	3.01	2.94	2.92	2.90
AVG	0.81	1.00	1.16	1.32	1.41	0.99	1.05	0.98	1.01

**Table 6:** The average mean errors for the total flow time criterion of the nine methods for each instance set.

Instances’ Group	Hybrid TROA	Hybrid GWO	Hybrid BA	Hybrid PSO	Hybrid ABC	Hybrid WOA	Hybrid TSO	Hybrid SSA	Hybrid FA
10 × 5	0.14	0.15	0.55	0.49	0.46	0.27	0.23	0.34	0.29
10 × 10	0.04	0.15	0.62	0.65	0.4	0.24	0.40	0.31	0.19
20 × 5	0.24	0.25	0.75	0.52	0.65	0.33	0.41	0.38	0.26
20 × 10	1.08	0.90	2.79	1.67	2.48	1.21	1.23	1.27	1.07
50 × 10	1.51	1.53	2.98	2.01	2.52	1.69	1.82	2.01	1.92
50 × 20	1.74	1.94	2.33	1.83	2.38	1.38	1.43	1.52	1.22
AVG	0.79	0.82	1.67	1.20	1.48	0.85	0.92	0.97	0.83

**Table 7:** Results for Friedman’s test, Friedman’s Aligned test, and the Quade test for the nine methods with the makespan criterion.

	Method	Friedmans Test	Friedmans Aligned Test	Quade Test
Ranking	Hybrid TROA	2.9583	130.7917	2.6586
	Hybrid WOA	4.2167	228.8500	4.1264
	Hybrid SSA	4.2417	215.5333	4.2189
	Hybrid GWO	4.5083	234.1167	4.4038
	Hybrid FA	4.5083	239.3667	4.4892
	Hybrid TSO	5.1667	272.5417	4.8939
	Hybrid BA	5.4083	315.6167	5.7370
	Hybrid PSO	6.9500	391.1333	6.8332
Measure	Hybrid ABC	7.0417	406.5500	7.6391
Statistic F		23.4799	23.9840	18.6070
*p*-value		0.0000	0.0000	0.0000

**Table 8:** Post hoc adjusted *p*-values (Bonferroni, Holm, FDR-BY, Sidak, FDR-BH, and Hommel) for comparisons between the Hybrid TROA and the competing algorithms after Friedman’s test for the makespan criterion.

	Bonferroni	Holm	FDR-BY	SIDAK	FDR-BH	Hommel
Hybrid SSA	0.0077	0.0019	0.0030	0.0076	0.0011	0.0012
Hybrid GWO	0.0006	0.0003	0.0003	0.0006	0.0001	0.0002
Hybrid TSO	0.0000	0.0000	0.0000	0.0000	0.0000	0.0000
Hybrid WOA	0.0096	0.0019	0.0033	0.0096	0.0012	0.0012
Hybrid FA	0.0006	0.0003	0.0003	0.0006	0.0001	0.0002
Hybrid PSO	0.0000	0.0000	0.0000	0.0000	0.0000	0.0000
Hybrid ABC	0.0000	0.0000	0.0000	0.0000	0.0000	0.0000
Hybrid BA	0.0000	0.0000	0.0000	0.0000	0.0000	0.0000

**Table 9:** Results for Friedman’s test, Friedman’s Aligned test, and the Quade test for the nine methods for total flow time criterion.

	Method	Friedman’s Test	Friedman’s Aligned Test	Quade Test
Ranking	Hybrid TROA	2.8333	147.3083	2.9249
	Hybrid GWO	2.9583	158.0833	2.9090
	Hybrid FA	3.8000	197.2167	3.6497
	Hybrid WOA	4.1000	209.7250	3.9552
	Hybrid TSO	4.6167	242.7833	4.4705
	Hybrid SSA	5.1000	260.7583	4.9251
	Hybrid PSO	6.3667	346.2000	6.1716
	Hybrid ABC	7.0000	413.3750	7.4119
Measure	Hybrid BA	8.2250	459.0500	8.5821
Statistic F		54.3484	52.1442	40.3494
*p*-value		0.0000	0.0000	0.0000

**Table 10:** Post hoc adjusted *p*-values (Bonferroni, Holm, FDR-BY, Sidak, FDR-BH, and Hommel) for comparisons between the Hybrid TROA and the competing algorithms after Friedman’s test for the total flow time criterion.

Hybrid TROA Vs.	Bonferroni	Holm	FDR-BY	SIDAK	FDR-BH	Hommel
Hybrid SSA	0.0000	0.0000	0.0000	0.0000	0.0000	0.0000
Hybrid GWO	1.0000	0.7256	1.0000	1.0000	0.7256	0.7256
Hybrid TSO	0.0000	0.0000	0.0000	0.0000	0.0000	0.0000
Hybrid WOA	0.0033	0.0012	0.0015	0.0033	0.0005	0.0012
Hybrid FA	0.0548	0.0137	0.0213	0.0535	0.0078	0.0137
Hybrid PSO	0.0000	0.0000	0.0000	0.0000	0.0000	0.0000
Hybrid ABC	0.0000	0.0000	0.0000	0.0000	0.0000	0.0000
Hybrid BA	0.0000	0.0000	0.0000	0.0000	0.0000	0.0000

**Table 11:** Comparisons of the AME of the Hybrid TROA against other nature-inspired meta-heuristics from the literature for each instance group.

Instances’ Group	Hybrid TROA	QABC	IABC	CWA	HAPSO	DDE	PSOENT	HMSA
10 × 5	0.00	0.04	0.57	0.00	0.00	0.46	0.00	0.85
10 × 10	0.24	0.01	0.75	0.67	0.09	0.93	0.07	1.59
20 × 5	0.27	0.01	0.66	0.68	0.07	0.79	0.08	0.88
20 × 10	0.06	0.01	0.14	0.08	0.05	0.17	0.02	0.41
50 × 10	1.52	0.53	1.79	0.79	2.01	2.26	2.11	1.88
50 × 20	2.76	1.04	2.74	2.38	3.2	3.11	3.83	1.7
AVG	0.81	0.27	1.11	0.77	0.90	1.29	1.02	1.22

**Table 12:** Energy-Efficient PFSP results for Hybrid TROA and Hybrid GWO Using Taillard’s Datasets.

			Hybrid TROA				Hybrid GWO					
Instance			$C_{max}^{TROA}$ (sec)	Energy (kJ)	Objective Z	Total kWh	$C_{max}^{GWO}$ (sec)	Energy (kJ)	Objective Z	Total kWh	%Δ $C_{max}$	%Δ*E*
N	n	m										
1	20	5	1072	14,259.65	0.8276	3.96	1063	14,259.65	0.8448	3.96	−0.85	0.00
2	20	5	1081	14,381.18	0.7929	3.99	1067	17,386.87	0.8838	4.83	−1.31	17.29
3	20	5	957	12,879.55	0.8452	3.58	956	12,879.55	0.8833	3.58	−0.10	0.00
4	20	5	1196	15,511.69	0.8966	4.31	1183	15,487.25	0.8822	4.30	−1.10	−0.16
5	20	5	1017	13,929.19	0.8022	3.87	1027	13,929.19	0.8952	3.87	0.97	0.00
6	20	5	1114	12,124.68	0.8928	3.37	1086	14,261.34	0.9036	3.96	−2.58	14.98
7	20	5	978	13,980.66	0.7704	3.88	978	13,980.66	0.8272	3.88	0.00	0.00
8	20	5	1085	14,390.91	0.872	4.00	1085	14,390.91	0.8743	4.00	0.00	0.00
9	20	5	1110	14,621.28	0.8487	4.06	1110	14,621.28	0.9031	4.06	0.00	0.00
10	20	5	983	13,172.18	0.8465	3.66	980	13,172.18	0.8655	3.66	−0.31	0.00
11	20	10	1108	39,305.09	0.6904	10.92	1069	39,521.74	0.784	10.98	−3.65	0.55
12	20	10	1296	40,802.07	0.8268	11.33	1306	40,515.70	0.8392	11.25	0.77	−0.71
13	20	10	1148	36,825.24	0.8184	10.23	1146	37,310.87	0.8389	10.36	−0.17	1.30
14	20	10	1012	34,055.30	0.7214	9.46	996	34,421.36	0.8559	9.56	−1.61	1.06
15	20	10	1039	36,021.69	0.7945	10.01	1050	35,735.31	0.789	9.93	1.05	−0.80
16	20	10	1021	35,281.64	0.7704	9.80	1000	35,062.50	0.8797	9.74	−2.10	−0.62
17	20	10	1029	35,347.50	0.7432	9.82	1026	35,347.50	0.8137	9.82	−0.29	0.00
18	20	10	1120	37,714.68	0.7744	10.48	1096	37,796.86	0.7944	10.50	−2.19	0.22
19	20	10	1096	39,094.96	0.7282	10.86	1063	39,528.03	0.8475	10.98	−3.10	1.10
20	20	10	1235	37,203.04	0.8176	10.33	1195	39,932.31	0.8409	11.09	−3.35	6.83
21	20	20	1736	70,412.23	0.8449	19.56	1662	72,250.92	0.8345	20.07	−4.45	2.54
22	20	20	1591	66,380.98	0.8068	18.44	1505	66,984.82	0.8485	18.61	−5.71	0.90
23	20	20	1846	57,988.12	0.8671	16.11	1685	71,593.68	0.8747	19.89	−9.55	19.00
24	20	20	1614	70,110.34	0.7923	19.48	1590	69,903.04	0.8244	19.42	−1.51	−0.30
25	20	20	1713	73,302.85	0.8263	20.36	1657	72,988.89	0.8585	20.27	−3.38	−0.43
26	20	20	1639	70,151.58	0.8227	19.49	1641	69,862.95	0.844	19.41	0.12	−0.41
27	20	20	1692	71,343.10	0.8386	19.82	1703	70,324.98	0.8653	19.53	0.65	−1.45
28	20	20	1724	68,406.53	0.8569	19.00	1627	70,963.62	0.8466	19.71	−5.96	3.60
29	20	20	1750	58,493.57	0.844	16.25	1644	72,866.75	0.8522	20.24	−6.45	19.73
30	20	20	1632	68,941.10	0.7943	19.15	1568	70,196.76	0.801	19.50	−4.08	1.79
31	50	5	2348	28,853.79	0.8497	8.01	2321	33,538.55	0.8698	9.32	−1.16	13.97
32	50	5	2582	36,241.06	0.8887	10.07	2582	36,241.06	0.9438	10.07	0.00	0.00
33	50	5	2308	33,330.65	0.862	9.26	2308	33,330.65	0.9229	9.26	0.00	0.00
34	50	5	2559	35,346.00	0.9003	9.82	2567	35,346.00	0.9293	9.82	0.31	0.00
35	50	5	2488	35,391.97	0.8584	9.83	2488	35,391.97	0.9635	9.83	0.00	0.00
36	50	5	2539	36,026.91	0.8877	10.01	2547	36,026.91	0.9137	10.01	0.31	0.00
37	50	5	2429	34,255.31	0.8734	9.52	2426	34,255.31	0.9283	9.52	−0.12	0.00
38	50	5	2543	34,328.26	0.9283	9.54	2547	34,328.26	0.9362	9.54	0.16	0.00
39	50	5	2283	32,338.69	0.8824	8.98	2283	32,338.69	0.9254	8.98	0.00	0.00
40	50	5	2507	35,362.94	0.8921	9.82	2507	35,362.94	0.9191	9.82	0.00	0.00
41	50	10	2635	80,904.96	0.8701	22.47	2609	93,205.05	0.882	25.89	−1.00	13.20
42	50	10	2558	83,931.23	0.8556	23.31	2558	83,851.81	0.8823	23.29	0.00	−0.09
43	50	10	2452	90,819.96	0.8167	25.23	2409	90,819.96	0.8576	25.23	−1.78	0.00
44	50	10	2637	95,014.17	0.8491	26.39	2588	94,381.24	0.8602	26.22	−1.89	−0.67
45	50	10	2721	80,963.17	0.8786	22.49	2698	93,345.70	0.8958	25.93	−0.85	13.27
46	50	10	2632	74,554.99	0.8552	20.71	2586	93,908.92	0.9029	26.09	−1.78	20.61
47	50	10	2632	94,266.42	0.8221	26.19	2600	93,877.27	0.8961	26.08	−1.23	−0.41
48	50	10	2568	73,331.41	0.8233	20.37	2488	92,834.76	0.8875	25.79	−3.22	21.01
49	50	10	2539	91,668.27	0.8353	25.46	2547	92,026.86	0.8724	25.56	0.31	0.39
50	50	10	2696	94,764.15	0.8501	26.32	2633	93,993.76	0.8824	26.11	−2.39	−0.82
51	50	20	3331	181,345.73	0.8556	50.37	3240	181,898.20	0.882	50.53	−2.81	0.30
52	50	20	3123	174,089.79	0.8387	48.36	3088	172,722.99	0.8723	47.98	−1.13	−0.79
53	50	20	3121	165,031.22	0.8395	45.84	3014	163,518.24	0.8496	45.42	−3.55	−0.93
54	50	20	3273	140,950.22	0.8638	39.15	3087	173,845.57	0.8454	48.29	−6.03	18.92
55	50	20	3079	171,780.71	0.8461	47.72	3088	172,223.30	0.8404	47.84	0.29	0.26
56	50	20	3156	140,659.29	0.8446	39.07	3003	174,133.02	0.8232	48.37	−5.09	19.22
57	50	20	3155	143,900.99	0.8301	39.97	3023	175,590.57	0.8337	48.78	−4.37	18.05
58	50	20	3173	167,398.52	0.8167	46.50	2975	173,472.07	0.8403	48.19	−6.66	3.50
59	50	20	3164	174,971.18	0.8085	48.60	3176	174,883.59	0.8565	48.58	0.38	−0.05
60	50	20	3150	178,333.28	0.8342	49.54	3151	178,177.72	0.8572	49.49	0.03	−0.09

## Data Availability

The data used in this study are publicly available and accessible through the following online repository: https://data.mendeley.com/datasets/5txxwj2g6b/1 (accessed on 22 February 2026).

## Appendix A

**Table A1:** Post hoc adjusted *p*-values (Bonferroni, Holm, FDR-BY, Sidak, FDR-BH, and Hommel) for the pairwise comparisons between the Hybrid TROA and the competing algorithms after Friedman’s Aligned Ranks test for the makespan criterion.

Hybrid TROA Vs.	Bonferroni	Holm	FDR-BY	SIDAK	FDR-BH	Hommel
Hybrid SSA	0.0235	0.0029	0.0080	0.0232	0.0029	0.0029
Hybrid GWO	0.0023	0.0009	0.0010	0.0023	0.0004	0.0009
Hybrid TSO	0.0000	0.0000	0.0000	0.0000	0.0000	0.0000
Hybrid WOA	0.0046	0.0012	0.0018	0.0046	0.0007	0.0012
Hybrid FA	0.0011	0.0006	0.0006	0.0011	0.0002	0.0006
Hybrid PSO	0.0000	0.0000	0.0000	0.0000	0.0000	0.0000
Hybrid ABC	0.0000	0.0000	0.0000	0.0000	0.0000	0.0000
Hybrid BA	0.0000	0.0000	0.0000	0.0000	0.0000	0.0000

**Table A2:** Post hoc adjusted *p*-values (Bonferroni, Holm, FDR-BY, Sidak, FDR-BH, and Hommel) for the pairwise comparisons between the Hybrid TROA and the competing algorithms after the Quade test for the makespan criterion.

Hybrid TROA Vs.	Bonferroni	Holm	FDR-BY	SIDAK	FDR-BH	Hommel
Hybrid SSA	0.0143	0.0036	0.0055	0.0142	0.0020	0.0033
Hybrid GWO	0.0039	0.0014	0.0017	0.0039	0.0006	0.0014
Hybrid TSO	0.0001	0.0000	0.0000	0.0001	0.0000	0.0000
Hybrid WOA	0.0262	0.0036	0.0089	0.0259	0.0033	0.0033
Hybrid FA	0.0020	0.0010	0.0011	0.0020	0.0004	0.0010
Hybrid PSO	0.0000	0.0000	0.0000	0.0000	0.0000	0.0000
Hybrid ABC	0.0000	0.0000	0.0000	0.0000	0.0000	0.0000
Hybrid BA	0.0000	0.0000	0.0000	0.0000	0.0000	0.0000

**Table A3:** Post hoc adjusted *p*-values (Bonferroni, Holm, FDR-BY, Sidak, FDR-BH, and Hommel) for the pairwise comparisons between the Hybrid TROA and the competing algorithms after Friedman’s Aligned Ranks test for the total flow time criterion.

Hybrid TROA Vs.	Bonferroni	Holm	FDR-BY	SIDAK	FDR-BH	Hommel
Hybrid SSA	0.0005	0.0003	0.0004	0.0005	0.0001	0.0003
Hybrid GWO	1.0000	0.7052	1.0000	0.9999	0.7052	0.7052
Hybrid TSO	0.0064	0.0032	0.0035	0.0064	0.0013	0.0032
Hybrid WOA	0.2276	0.0853	0.1031	0.2062	0.0379	0.0853
Hybrid FA	0.6382	0.1596	0.2478	0.4858	0.0912	0.1596
Hybrid PSO	0.0000	0.0000	0.0000	0.0000	0.0000	0.0000
Hybrid ABC	0.0000	0.0000	0.0000	0.0000	0.0000	0.0000
Hybrid BA	0.0000	0.0000	0.0000	0.0000	0.0000	0.0000

**Table A4:** Post hoc adjusted *p*-values (Bonferroni, Holm, FDR-BY, Sidak, FDR-BH, and Hommel) for the pairwise comparisons between the Hybrid TROA and the competing algorithms after the Quade test for the total flow time criterion.

Hybrid TROA Vs.	Bonferroni	Holm	FDR-BY	SIDAK	FDR-BH	Hommel
Hybrid SSA	0.0001	0.0000	0.0000	0.0001	0.0000	0.0000
Hybrid GWO	1.0000	0.9716	1.0000	1.0000	0.9716	0.9716
Hybrid TSO	0.0044	0.0022	0.0024	0.0044	0.0009	0.0022
Hybrid WOA	0.1672	0.0627	0.0757	0.1555	0.0279	0.0627
Hybrid FA	0.8294	0.2073	0.3220	0.5834	0.1185	0.2073
Hybrid PSO	0.0000	0.0000	0.0000	0.0000	0.0000	0.0000
Hybrid ABC	0.0000	0.0000	0.0000	0.0000	0.0000	0.0000
Hybrid BA	0.0000	0.0000	0.0000	0.0000	0.0000	0.0000
